# Inhibition of AKT/GSK3β/CREB Pathway Improves the Responsiveness to AMPA Receptor Antagonists by Regulating GRIA1 Surface Expression in Chronic Epilepsy Rats

**DOI:** 10.3390/biomedicines9040425

**Published:** 2021-04-14

**Authors:** Ji-Eun Kim, Duk-Shin Lee, Hana Park, Tae-Hyun Kim, Tae-Cheon Kang

**Affiliations:** Department of Anatomy and Neurobiology, Institute of Epilepsy Research, College of Medicine, Hallym University, Chuncheon 24252, Korea; jieunkim@hallym.ac.kr (J.-E.K.); dslee84@hallym.ac.kr (D.-S.L.); M19050@hallym.ac.kr (H.P.); hyun1028@hallym.ac.kr (T.-H.K.)

**Keywords:** 3CAI, GRIA1, GRIA2, intractable epilepsy, PICK1, protein kinase C

## Abstract

α-Amino-3-hydroxy-5-methylisoxazole-4-propionic acid receptor (AMPAR) has been reported as one of the targets for treatment of epilepsy. Although maladaptive regulation of surface expression of glutamate ionotropic receptor AMPA type subunit 1 (GRIA1) subunit is relevant to the responsiveness to AMPAR antagonists (perampanel and GYKI 52466) in LiCl-pilocarpine-induced chronic epilepsy rats, the underlying mechanisms of refractory seizures to AMPAR antagonists have yet been unclear. In the present study, we found that both AMPAR antagonists restored the up-regulations of GRIA1 surface expression and Src family-mediated glycogen synthase kinase 3β (GSK3β)-Ca^2+^/cAMP response element-binding protein (CREB) phosphorylations to control levels in responders (whose seizure activities were responsive to AMPAR) but not non-responders (whose seizure activities were uncontrolled by AMPAR antagonists). In addition, 3-chloroacetyl indole (3CAI, an AKT inhibitor) co-treatment attenuated spontaneous seizure activities in non-responders, accompanied by reductions in AKT/GSK3β/CREB phosphorylations and GRIA1 surface expression. Although AMPAR antagonists reduced GRIA2 tyrosine (Y) phosphorylations in responders, they did not affect GRIA2 surface expression and protein interacting with C kinase 1 (PICK1) protein level in both responders and non-responders. Therefore, our findings suggest that dysregulation of AKT/GSK3β/CREB-mediated GRIA1 surface expression may be responsible for refractory seizures in non-responders, and that this pathway may be a potential target to improve the responsiveness to AMPAR antagonists.

## 1. Introduction

Epilepsy is a common clinical neurological disease characterized by spontaneous seizures due to abnormal neuronal discharges. Over decades, the incidence of epilepsy presents an increasing tendency with a reported number of 65 million patients worldwide. About 30% temporal lobe epilepsy (TLE) patients show refractory seizures that are uncontrolled by standard medications with antiepileptic drugs (AEDs). In addition, patients with intractable seizures have a high mortality rate [1,2]. Given the inefficacy of AEDs and the unrelenting nature of intractable epilepsy, therefore, the necessity to explore the underlying mechanisms of generation of refractory seizures is undoubted.

Although the pathogenesis of epilepsy remains to be fully elucidated, hyper-activation of the glutamate receptor is one of the important causes of epilepsy. A massive release of glutamate and the subsequent over-activation of glutamate receptors cause aberrant neuronal hyper-excitability, followed by delayed neuronal death and secondary injury [3,4]. Recently, α-amino-3-hydroxy-5-methylisoxazole-4-propionic acid receptor (AMPAR) has been focused on as one of the major therapeutic targets for treatment of epilepsy [5] since AMPAR antagonists, but not N-methyl-D-aspartate receptor (NMDAR) antagonists, terminate status epilepticus (SE, a prolonged seizure activity) and seizure activity in animal models and preferentially suppress seizure-related long-term consequences [6,7,8].

AMPAR is one of the ionotropic glutamate receptors, which exists as a homomeric or heteromeric assembly of four subunits (glutamate ionotropic receptor AMPA type subunit (GRIA) 1–4) encoded by different genes [9]. GRIA subunits preferentially dimerize soon after the translocation in the endoplasmic reticulum (ER) and tetramerize prior to ER exit [10]. Activity-dependent phosphorylation/dephosphorylation of intracellular C-terminal sites on the AMPAR subunits GRIA1 and GRIA2 regulate their synaptic trafficking and functional properties [11,12]: Phosphorylation of GRIA1 at serine (S) 831 and/or S845 sites increases both open channel conductance and its surface expression [13,14]. In contrast, GRIA2 S880 phosphorylation facilitates its internalization [15]. Interestingly, GRIA2 subunit regulates the ion-permeable properties of AMPAR. GRIA2-containing AMPAR is permeable to Na^+^ and K^+^ but not Ca^2+^. In contrast, GRIA2-lacking AMPAR is highly permeable to Ca^2+^, which activates a series of intracellular protein kinases or immediate early genes. In the hippocampus of epileptic animals, GRIA2 surface expression is decreased [16,17], while >95% of AMPARs contain the GRIA2 subunit under physiological conditions [18]. Therefore, it is likely that increased membrane GRIA1/GRIA2 ratio may be an important component of the ictogenesis and the seizure-associated consequences by leading to a high Ca^2+^ permeability of AMPAR in the epileptic brain. Indeed, we have reported that membrane GRIA1/GRIA2 ratio is significantly higher in chronic epilepsy rats than that in control animals, indicating the existing preponderance of Ca^2+^-permeable AMPAR, although both GRIA1 and GRIA2 levels are lower in the hippocampus. In addition, AMPAR antagonists (perampanel and GYKI 52466) decrease membrane GRIA1/GRIA2 ratio by reducing GRIA1, but not GRIA2, surface expression [19,20]. These effects of AMPAR antagonists are relevant to Src family/casein kinase 2 (CK2)/phosphatase and tensin homolog deleted on chromosome ten (PTEN)-mediated Ca^2+^/cAMP response element-binding protein (CREB) S133 phosphorylation, which are observed in responders (whose seizure activities are responsive to AMPA antagonists), but not non-responders (whose seizure activities are not uncontrolled) [19]. However, the up-stream signaling pathways regulating membrane GRIA1/GRIA2 ratio in the epileptic hippocampus have yet been elusive.

On the other hand, GRIA2 phosphorylations regulate their surface expression through interactions with glutamate receptor interacting protein 1 (GRIP1) and protein interacting with C kinase 1 (PICK1) that contains the postsynaptic density-95/Discs large/zona occludens-1 (PDZ) domain. GRIP1 enhances GRIA2 surface expression, whereas PICK1 facilitates its internalization [21,22]. Src family-mediated GRIA2 phosphorylations on tyrosine (Y) 869, Y873, and Y876 sites destabilize its interaction with GRIP1 and allow more GRIA2–PICK1 binding to promote its internalization [23,24]. In addition, protein kinase C (PKC)-mediated S880 phosphorylation also decreases GRIA2 binding to GRIP1, but not to PICK1, and results in rapid GRIA2 internalization [21]. However, the regulations of the GRIA2 subunit phosphorylations in response to AMPAR antagonists in the epileptic hippocampus are largely unknown. In an effort to understand the pathogenesis of intractable epilepsy, therefore, it will be noteworthy to more specifically investigate the underlying mechanisms of refractory seizures to AMPAR antagonists.

Here, we demonstrated that the anti-convulsive effects of AMPAR antagonists were closely relevant to the regulation of AKT/glycogen synthase kinase 3β (GSK3β)/CREB-mediated GRIA1 surface expression rather than the modulation of PICK1–GRIA2 internalization. In addition, impairment of this signaling pathway resulted in refractory seizures in response to AMPAR antagonists, which were improved by 3-chloroacetyl indole (3CAI, an AKT inhibitor) co-treatment. Therefore, our findings suggest that AKT/GSK3β/CREB pathway may be a potential therapeutic strategy to improve the treatment of intractable TLE in response to AMPAR antagonists.

## 2. Materials and Methods

### 2.1. Experimental Animals and Chemicals

Male Sprague–Dawley (SD) rats (7 weeks old) were provided with a commercial diet and water ad libitum under controlled temperature, humidity, and lighting conditions (22 ± 2 °C, 55 ± 5% and a 12:12 light/dark cycle with lights). Animal protocols were approved by the Institutional Animal Care and Use Committee of Hallym University (Hallym 2018-2, 26 April 2018 and Hallym 2018-21, 8 June 2018). All reagents were purchased from Sigma-Aldrich (St. Louis, MO, USA), except as noted.

### 2.2. Generation of Chronic Epilepsy Rats

**Figure 1:**
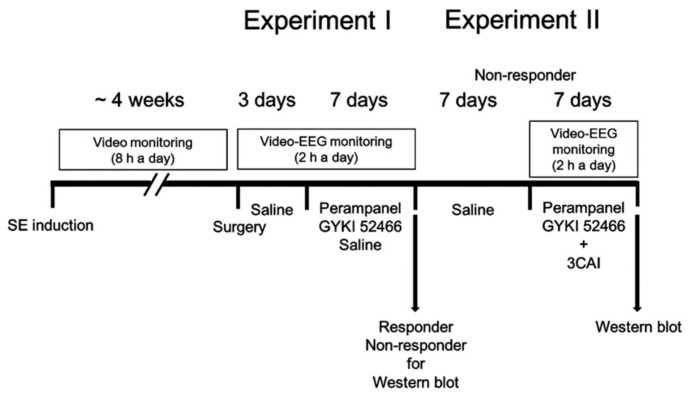
Scheme of the experimental design in the present study.

Figure 1 illustrates the design of the drug trial methodology, which was a modified protocol based on Ko and Kang [25]. Animals were intraperitoneally (i.p.) given LiCl (127 mg/kg) 24 h before the pilocarpine treatment. Animals were treated with pilocarpine (30 mg/kg, i.p.) 20 min after atropine methylbromide (5 mg/kg i.p.). Two hours after SE on-set, animals were administered diazepam (Valium; Hoffman la Roche, Neuilly sur-Seine, France; 10 mg/kg, i.p.) and dosage was repeated, as needed. Control animals received saline in place of pilocarpine. Animals were video-monitored 8 h a day for general behavior and occurrence of spontaneous seizures by 4 weeks after SE induction. Behavioral seizure severity was evaluated according to Racine’s scale [26]: 1, immobility, eye closure, twitching of vibrissae, sniffing, facial clonus; 2, head nodding associated with more severe facial clonus; 3, clonus of one forelimb; 4, rearing, often accompanied by bilateral forelimb clonus; and 5, rearing with loss of balance and falling accompanied by generalized clonic seizures. We classified chronic epilepsy rats that showed behavioral seizures with seizure score ≥3 more than once.

### 2.3. Surgery

Control and epilepsy rats were implanted with monopolar stainless steel electrodes (Plastics One, Roanoke, VA, USA) in the right hippocampus (stereotaxic coordinates were −3.8 mm posterior; 2.0 mm lateral; −2.6 mm depth to bregma) under Isoflurane anesthesia (3% induction, 1.5–2% for surgery, and 1.5% maintenance in a 65:35 mixture of N_2_O:O_2_). Animals were also implanted with a brain infusion kit 1 (Alzet, Cupertino, CA, USA) to infuse with 3CAI (an AKT inhibitor, 25 μM) into the right lateral ventricle (1 mm posterior; 1.5 mm lateral; −3.5 mm depth to the bregma, see below). Throughout surgery, core temperature of each rat was maintained at 37–38 °C. Electrode was secured to the exposed skull with dental acrylic.

### 2.4. Drug Trials, EEG Analysis and Quantification of Behavioral Seizure Activity

#### 2.4.1. Experiment I

After baseline seizure activity was determined over 3 days, perampanel (2-(2-oxo-1-phenyl-5-pyridin-2-yl-1,2-dihydropyridin-3-yl)benzonitrile; 8 mg/kg, i.p, Eisai Korea Inc, Seoul, South Korea), GYKI 52466 (1-(4-aminophenyl)-4-methyl-7,8-methylenedioxy-5H-2,3-benzodiazepine hydrochloride, 10 mg/kg, i.p.) or saline (vehicle) was daily administered at 6:00 p.m. over a 1-week period [19,20,27]. Electroencephalographic (EEG) signals were detected with a DAM 80 differential amplifier (0.1–3000 Hz bandpass; World Precision Instruments, Sarasota, FL, USA) 2 h a day at the same time over a 1-week period. The data were digitized and analyzed using LabChart Pro v7 (ADInstruments, Bella Vista, New South Wales, Australia). Behavioral seizure severity was measured according to Racine’s scale [26], as aforementioned. Non-responders were defined as showing no reduction in total seizure frequency in a 7-day period, as compared with the pre-treatment stage. After recording (18 h after the last drug treatment), animals were used for Western blot.

#### 2.4.2. Experiment II

Some non-responders in experiment I were given saline (i.p.) over a 7-day period. Thereafter, animals were daily given perampanel or GYKI 52466 by the aforementioned method. Rats were also connected with Alzet 1007D osmotic pump (Alzet, Cupertino, CA, USA) containing vehicle or 3CAI (25 μM) [28]. The pump was placed in a subcutaneous pocket in the dorsal region. In a pilot study and our previous study [28], this dosage of 3CAI did not show behavioral and neurological defects and could not change the seizure susceptibility and seizure severity in response to pilocarpine. After recording (18 h after the last drug treatment), animals were used for Western blot.

### 2.5. Western Blot

Animals were sacrificed by decapitation, and their hippocampi were obtained and homogenized in lysis buffer containing protease inhibitor cocktail (Roche Applied Sciences, Branford, CT, USA) and phosphatase inhibitor cocktail (PhosSTOP^®^, Roche Applied Science, Branford, CT, USA). Thereafter, total protein concentration calibrated using a Micro BCA Protein Assay Kit (Pierce Chemical, Rockford, IL, USA). To analyze membrane GRIA subunit expressions, the bilateral hippocampal tissues (~100 mg) were washed gently with ice-cold PBS and chopped and homogenized for 10 strokes using a Dounce tissue homogenizer. Thereafter, membrane proteins were extracted with a subcellular Protein Fractionation Kit for Tissues (Thermo Scientific Korea, Seoul, South Korea), according to the manufacturer’s instructions. Western blot was performed by the standard protocol: Sample proteins (10 μg) were separated on a Bis-Tris sodium dodecyl sulfate-poly-acrylamide gel (SDS-PAGE) and transferred to membranes. Membranes were incubated with 2% bovine serum albumin (BSA) in Tris-buffered saline (TBS; in mM 10 Tris, 150 NaCl, pH 7.5, and 0.05% Tween 20), and then reacted with primary antibodies (Table 1) overnight at 4 °C. After washing, membranes were incubated in a solution containing horseradish peroxidase (HRP)-conjugated secondary antibodies for 1 h at room temperature. Immunoblots were detected and quantified using ImageQuant LAS4000 system (GE Healthcare Korea, Seoul, South Korea). Optical densities of proteins were calculated with the corresponding amount of β-actin or N-cadherin.

### 2.6. Data Analysis

Seizure parameters (frequency, duration and Racine scores) were assessed by different investigators who were blind to the classification of animal groups and treatments. Shapiro–Wilk *W*-test was used to evaluate the values on normality. Mann–Whitney U-test, Wilcoxon signed rank test, Student’s *t*-test, and paired Student’s *t*-test were applied to determine statistical significance of data. Comparisons among groups were also performed using repeated measures ANOVA, Friedman test, and one-way ANOVA followed by Bonferroni’s post hoc comparisons. A *p*-value less than 0.05 was considered to be significant.

## 3. Results

### 3.1. Effects of AMPAR Antagonists on Chronic Spontaneous Seizure Activity

**Figure 2:**
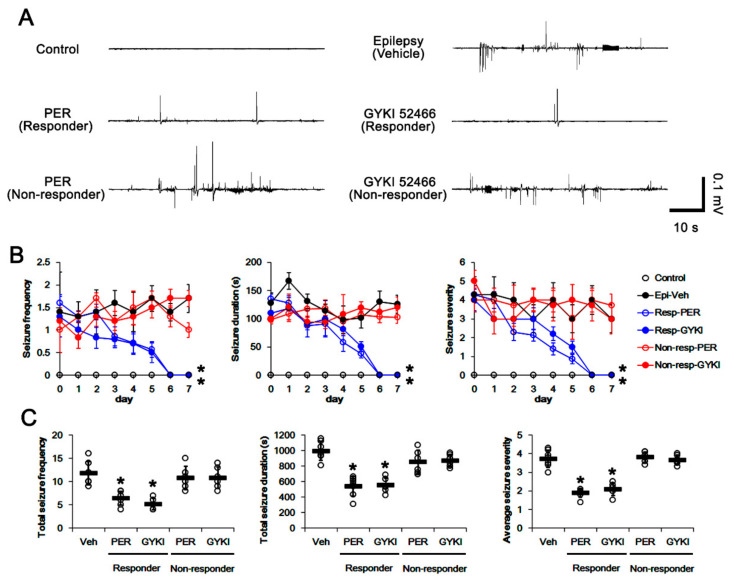
The effects of perampanel (PER) and GYKI 52466 (GYKI) on spontaneous seizure activities in chronic epilepsy rats. Both α-amino-3-hydroxy-5-methylisoxazole-4-propionic acid receptor (AMPAR) antagonists effectively attenuate spontaneous seizure activities in responders. (**A**) Representative electroencephalograms (EEG) in each group at 2 days after treatment. (**B**) Quantitative analyses of the chronological effects of AMPAR antagonists on seizure frequency, seizure duration, and seizure severity (seizure score) over 7-day period. Error bars indicate SD (* p < 0.05 vs. vehicle (Veh)-treated animals; Friedman test for seizure frequency and seizure severity; Repeated measures ANOVA for seizure duration). (**C**) Quantitative analyses of seizure frequency, total seizure duration and average behavioral seizure score (seizure severity) in 7-day period. Open circles indicate each individual value. Horizontal bars indicate mean value. Error bars indicate SD (* p < 0.05 vs. vehicle (Veh)-treated animals; Mann–Whitney U-test for seizure frequency and seizure severity; Student *t*-test for seizure duration).

In epileptic rats, the total seizure frequency (number of seizures), the total electroencephalographic (EEG) seizure duration, and average seizure severity (behavioral seizure core) were 11.9 ± 2.5, 996.4 ± 121 s, and 3.7 ± 0.5 over 1-week period, respectively (*n* = 7, Figure 2A–C). In responders, perampanel gradually reduced seizure frequency (*χ*^2^_(1)_ = 5.5, *p* = 0.019, Friedman test, *n* = 7), seizure duration (*F*_(1,12)_ = 6.6, *p* = 0.025, repeated measures ANOVA, *n* = 7), and the seizure severity (*χ*^2^_(1)_ = 5.3, *p* = 0.021, Friedman test, *n* = 7) over 1-week period (Figure 2A,B). The total seizure frequency was 6.42 ± 1.5 (Z = 3.14, *p* = 0.001 vs. vehicle, Mann–Whitney U-test, *n* = 7), the total seizure duration was 538.3 ± 127 s (*t*_(12)_ = 13.43, *p* < 0.001 vs. vehicle, Student *t*-test, *n* = 7), and the average seizure severity was 1.9 ± 0.2 over 1-week period (Z = 2.89, *p* = 0.002 vs. vehicle, Mann–Whitney U-test, *n* = 7; Figure 2C). Six out of thirteen rats in perampanel-treated group were identified as non-responders whose seizure activities were uncontrolled by perampanel (total seizure frequency, 10.8 ± 2.5; total seizure duration, 852.2 ± 150.5 s; average seizure severity, 3.8 ± 0.3; Figure 2B,C).

GYKI 52466 also decreased seizure frequency (*χ*^2^_(1)_ = 6.2, *p* = 0.013, Friedman test, *n* = 6), total seizure duration (*F*_(1,11)_ = 5.9, *p* = 0.033, repeated measures ANOVA, *n* = 6), and the seizure severity (*χ*^2^_(1)_ = 4.8, *p* = 0.029, Friedman test, *n* = 6) in responders over 1-week period (Figure 2A,B). In responders to GYKI 52466, the total seizure frequency was 5.2 ± 1.2 (Z = 3.01, *p* = 0.001 vs. vehicle, Mann–Whitney U-test, *n* = 6), the total seizure duration was 551.7 ± 92.9 s (*t*_(11)_ = 10.01, *p* < 0.001 vs. vehicle, Student *t*-test, *n* = 6), and the average seizure severity was 2.1 ± 0.3 over 1-week period (Z = 2.86, *p* = 0.002 vs. vehicle, Mann–Whitney U-test, *n* = 6; Figure 2C). Six out of twelve rats in GYKI 52466-treated group were identified as non-responders (total seizure frequency, 10.8 ± 2.3; total seizure duration, 870.8 ± 80.5 s; average seizure severity, 3.7 ± 0.3; Figure 2B,C).

### 3.2. Effects of AMPAR Antagonists on GRIA2 Phosphorylations

**Figure 3:**
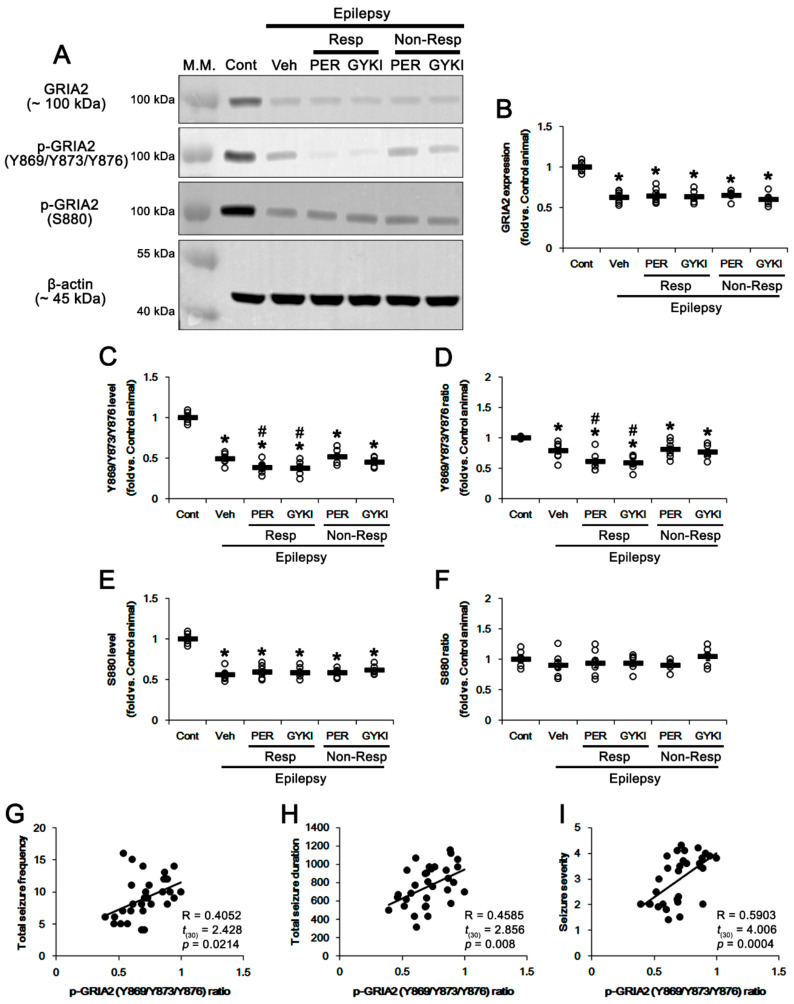
The effects of perampanel (PER) and GYKI 52466 (GYKI) on total glutamate ionotropic receptor AMPA type subunit 2 (GRIA2) expression and its phosphorylations in chronic epilepsy rats. (**A**) Representative images for Western blot of GRIA2 and its phosphorylation levels in the hippocampal tissues. (**B**–**F**) Quantifications of GRIA2 (**B**), p-GRIA2 Y869/Y873/Y876 (**C**), p-GRIA2 Y869/Y873/Y876/GRIA2 ratio (**D**), p-GRIA2 S880 (**E**), and p-GRIA2 S880/GRIA2 ratio (**F**) in the hippocampal tissues. Open circles indicate each individual value. Horizontal bars indicate mean value. Error bars indicate SEM (*, ^#^ *p* < 0.05 vs. control (Cont) and vehicle (Veh)-treated animals, respectively; one-way ANOVA with post hoc Bonferroni’s multiple comparison). (**G**–**I**) Linear regression analyses between p-GRIA2 Y869/Y873/Y876/GRIA2 ratio and total seizure frequency (**G**), total seizure duration (**H**), and seizure severity (**I**) in chronic epilepsy rats.

The phosphorylations of GRIA2 regulate its surface expression and influence AMPAR functionality [15,21,22,23,24]. Therefore, we first explored whether AMPAR antagonists affect GRIA2 protein expression and its phosphorylation levels in the epileptic hippocampus. Consistent with previous studies [16,17], total GRIA2 protein level was 37% lower in the epileptic hippocampus (*t*_(12)_ = 10.0, *p* < 0.001 vs. control animals, Student *t*-test; Figure 3A,B and Figure S1), as compared to control animals. Compatible to its protein level, GRIA2 Y869/Y873/Y876 phosphorylation levels were decreased to 0.49-fold of control level in the vehicle-treated epilepsy rats (*t*_(12)_ = 14.8, *p* < 0.001 vs. control animals, Student *t*-test; Figure 3A,C). GRIA2 Y phosphorylation ratio was 0.79-fold of control level in the vehicle-treated epilepsy rats (*t*_(12)_ = 3.8, *p* < 0.001 vs. control animals, Student *t*-test; Figure 3A,D).

In responders to perampanel and GYKI 52466, GRIA2 Y869/Y873/Y876 phosphorylation levels were significantly reduced to 0.39- and 0.38-fold of control levels without affecting GRIA2 protein levels, respectively (*F*_(2,17)_ = 4.8, *p* = 0.02 vs. vehicle, one-way ANOVA; Figure 3A–C and Figure S1). Thus, its Y phosphorylation ratios were 0.61- and 0.6-fold of control levels, respectively (*F*_(2,17)_ = 4.1, *p* = 0.04 vs. vehicle, one-way ANOVA; Figure 3A,D). In non-responders to perampanel and GYKI 52466, GRIA2 protein level and its Y869/Y873/Y876 phosphorylation levels/ratios were unaffected by each compound (Figure 3A–D). Similar to Y869/Y873/Y876 phosphorylation level, GRIA2 S880 phosphorylation level was reduced to 0.56-fold of control level in the vehicle-treated epilepsy rats (*t*_(12)_ = 12.6, *p* < 0.001 vs. control animals, Student *t*-test; Figure 3A,E and Figure S1). Thus, S880 phosphorylation ratio was 0.91-fold of control level in the vehicle-treated epilepsy rats (*t*_(12)_ = 1.2, *p* = 0.26 vs. control animals, Student *t*-test; Figure 3A,F). In responders, GRIA2 S880 phosphorylation level (*F*_(2,17)_ = 0.4, *p* = 0.65 vs. vehicle, one-way ANOVA; Figure 3A,E) and its ratio (*F*_(2,17)_ = 0.08, *p* = 0.92 vs. vehicle, one-way ANOVA; Figure 3A,F) were unaffected by perampanel and GYKI 52466. In non-responders, both perampanel and GYKI 52466 did not alter GRIA2 S880 phosphorylation level/ratio (Figure 3A,E,F). In addition, we analyzed the correlation between GRIA2 Y phosphorylation ratio and seizure parameters in chronic epilepsy rats. Linear regression analysis showed a direct proportional relationship between GRIA2 Y phosphorylation ratio and total seizure frequency with linear correlation coefficients of 0.4052 (*t*_(30)_ = 2.428, *p* = 0.0214; Figure 3G). The GRIA2 Y phosphorylation ratio also showed direct proportional relationships with total seizure duration (linear correlation coefficients, 0.4585; *t*_(30)_ = 2.856, *p* = 0.008; Figure 3H) and seizure severity (linear correlation coefficients, 0.5903; *t*_(30)_ = 4.006, *p* = 0.0004; Figure 3I). These findings indicate that anti-epileptic effects of AMPAR antagonists may be correlated with GRIA2 Y869/Y873/Y876 phosphorylations.

### 3.3. Effects of AMPAR Antagonists on PKC and Src Phosphorylations

Src family and PKC phosphorylate GRIA2 at Y869/Y873/Y876 and S880 sites, respectively [21,23,24]. Therefore, we confirmed whether both AMPAR antagonists affect GRIA2 phosphorylations through PKC and/or Src family-mediated signaling pathways.

**Figure 4:**
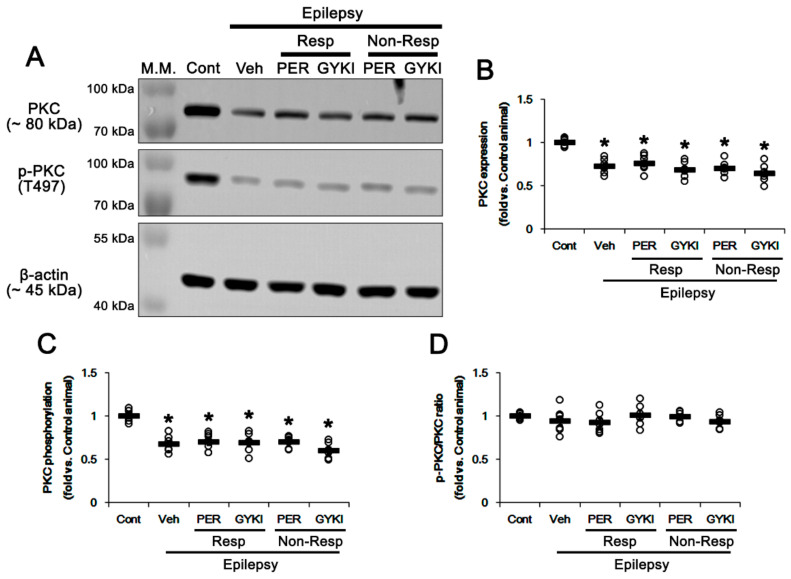
The effects of perampanel (PER) and GYKI 52466 (GYKI) on total protein kinase C (PKC) expression and its threonine (T) 497 phosphorylation in chronic epilepsy rats. (**A**) Representative images for western blot of PKC and its T497 phosphorylation level in the hippocampal tissues. (**B**–**D**) Quantifications of PKC (**B**), p-PKC T497 (**C**), and p-PKC/PKC ratio (**D**) in the hippocampal tissues. Open circles indicate each individual value. Horizontal bars indicate mean value. Error bars indicate SEM (* p < 0.05 vs. control animals; one-way ANOVA with post hoc Bonferroni’s multiple comparison).

In epileptic rats, PKC expression and its phosphorylation level were decreased to 0.72- (*t*_(12)_ = 7.9, *p* < 0.001 vs. control animals, Student *t*-test; Figure 4A,B and Figure S2) and 0.68-fold (*t*_(12)_ = 7.6, *p* < 0.001 vs. control animals, Student *t*-test; Figure 4A,C) of control levels, respectively. However, PKC phosphorylation ratio was similar to that in controls (*t*_(12)_ = 0.97, *p* = 0.35 vs. control animals, Student *t*-test; Figure 4A,D). Both perampanel and GYKI 52466 did not affect PKC protein level (*F*_(4,27)_ = 1.4, *p* = 0.26 vs. vehicle, one-way ANOVA), PKC phosphorylation (*F*_(4,27)_ = 1.1, *p* = 0.36 vs. vehicle, one-way ANOVA), and its phosphorylation ratio (*F*_(4,27)_ = 0.7, *p* = 0.58 vs. vehicle, one-way ANOVA) in non-responders as well as responders (Figure 4A–D and Figure S2).

**Figure 5:**
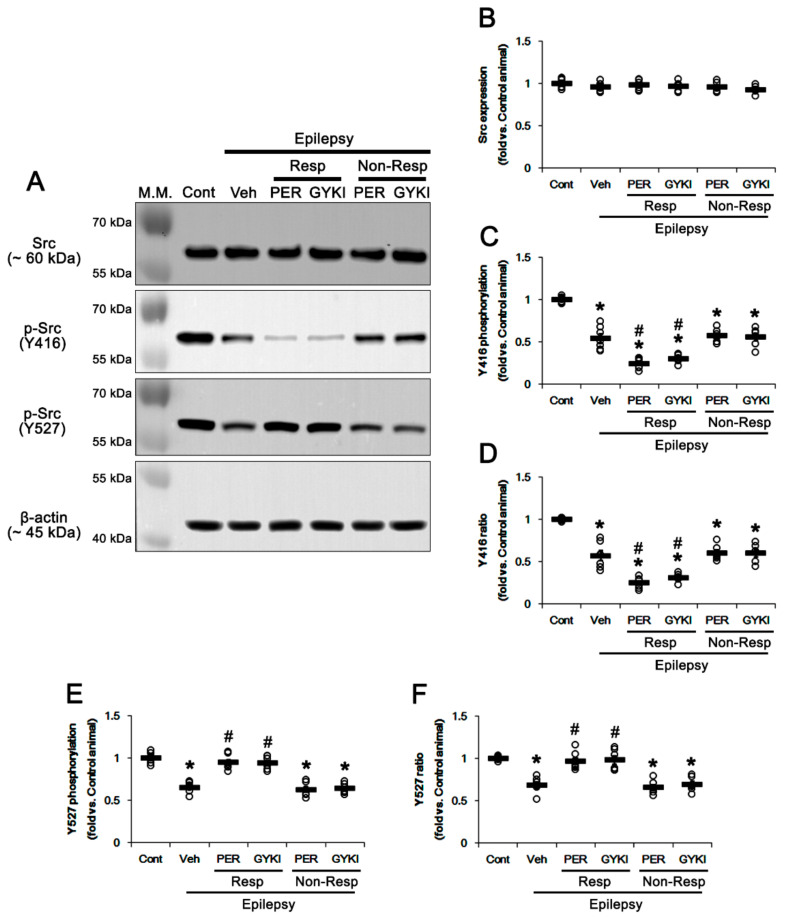
The effects of perampanel (PER) and GYKI 52466 (GYKI) on Src family and its phosphorylations in chronic epilepsy rats. (**A**) Representative images for Western blot of Src family and its phosphorylation levels in the hippocampal tissues. (**B**–**F**) Quantifications of Src2 (**B**), p-Src Y416 (**C**), p-Src Y416/Src ratio (**D**), p-Src Y527 (**E**), and p-Src Y527/GRIA2 ratio (**F**) in the hippocampal tissues. Open circles indicate each individual value. Horizontal bars indicate mean value. Error bars indicate SEM (*, ^#^ *p* < 0.05 vs. control (Cont) and vehicle (Veh)-treated animals, respectively; one-way ANOVA with post hoc Bonferroni’s multiple comparison).

In chronic epilepsy rats, Src family protein level was similarly observed, as compared to controls. Both AMPAR antagonists did not affect it (*F*_(4,27)_ = 0.9, *p* = 0.49, one-way ANOVA; Figure 5A,B and Figure S3). Src Y416 phosphorylation level was 0.54-fold lower in the epileptic hippocampus than that in controls (*t*_(12)_ = 8.6, *p* < 0.001 vs. control animals, Student *t*-test; Figure 5A,C) and its phosphorylation ratio was 0.57-fold of control level (*t*_(12)_ = 7.4, *p* < 0.001 vs. control animals, Student *t*-test; Figure 5A,D). In responders, perampanel and GYKI 52466 decreased Src Y416 phosphorylation level to 0.25- and 0.3-fold of control level, respectively (*F*_(2,17)_ = 19.6, *p* < 0.001 vs. vehicle, one-way ANOVA; Figure 5A,C and Figure S3). Both perampanel and GYKI 52466 reduced Y416 phosphorylation ratios to 0.25- and 0.31-fold of control level, respectively (*F*_(2,17)_ = 19.3, *p* < 0.001 vs. vehicle, one-way ANOVA; Figure 5A,D). In non-responders, both AMPAR antagonists did not affect Y416 phosphorylation level (*F*_(2,16)_ = 0.11, *p* = 0.89 vs. vehicle, one-way ANOVA; Figure 5A,C) and its ratio (*F*_(2,15)_ = 0.14, *p* = 0.87, one-way ANOVA; Figure 5A,D). In the epileptic hippocampus, Src Y527 phosphorylation level was also decreased to 0.65-fold of control level (*t*_(12)_ = 10.1, *p* < 0.001 vs. control animals, one-way ANOVA, Student *t*-test; Figure 5A,E and Figure S3). Y527 phosphorylation ratio was 0.69-fold of control level (*t*_(12)_ = 8.9, *p* < 0.001, Student *t*-test; Figure 5A,F). In responders, perampanel and GYKI 52466 recovered Src Y527 phosphorylation level (*F*_(2,17)_ = 31.1, *p* < 0.001 vs. vehicle, one-way ANOVA) and its phosphorylation ratio to control level (*F*_(2,17)_ = 16.5, *p* < 0.001 vs. vehicle, one-way ANOVA), respectively (Figure 5A,E,F). In non-responders, both AMPAR antagonists did not affect Y527 phosphorylation level (*F*_(2,16)_ = 0.22, *p* = 0.81 vs. vehicle, one-way ANOVA; Figure 5A,E) and its ratio (*F*_(2,16)_ = 0.3, *p* = 0.74, one-way ANOVA; Figure 5A,F). Src family activities are reversely modulated by phosphorylation of two distinct tyrosine sites: Y416 autophosphorylation upregulates Src kinase activity. In contrast, Y527 phosphorylation inhibits its activity, although dephosphorylation at this site cannot fully activate it [29,30]. Therefore, these findings indicate that refractory seizure activity to AMPAR antagonists may be relevant to dysregulated Src activity rather than PKC phosphorylation in responders.

### 3.4. Effects of AMPAR Antagonists on PICK1 Expression

**Figure 6:**
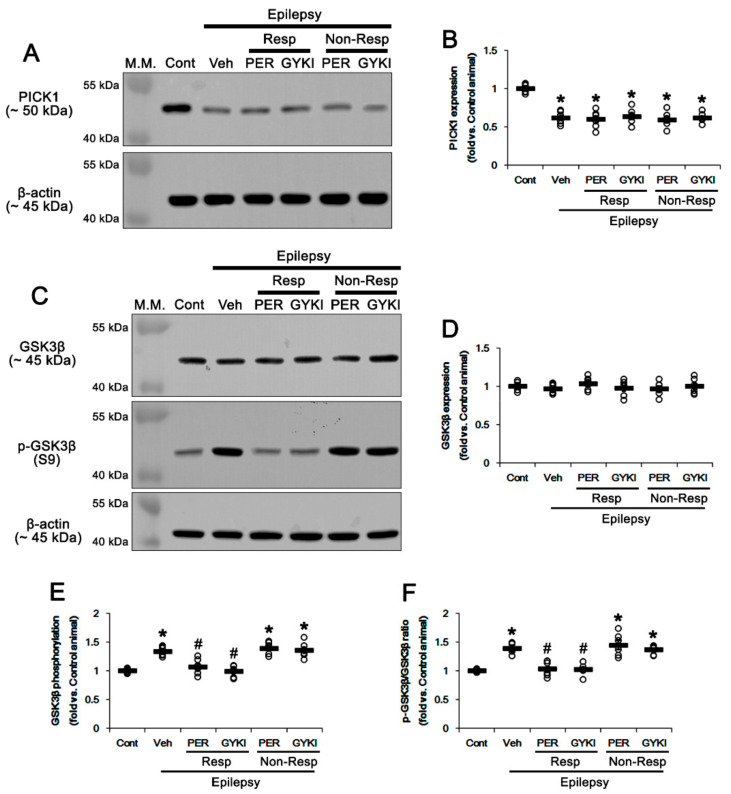
The effects of perampanel (PER) and GYKI 52466 (GYKI) on protein interacting with C kinase 1 (PICK1) expression and glycogen synthase kinase 3β (GSK3β) expression and its phosphorylation in chronic epilepsy rats. (**A**,**B**) Representative images for Western blot of PICK1 expression (**A**) and quantifications of PICK1 expression (**B**) in the hippocampal tissues. (**C**–**F**) Representative images for Western blot of GSK3β expression and its phosphorylation (**C**) and quantifications of GSK3β (**D**), p-GSK3β S9 (**E**), and p-GSK3β S9/GSK3β ratio (**F**) in the hippocampal tissues. Open circles indicate each individual value. Horizontal bars indicate mean value. Error bars indicate SEM (*, ^#^ *p* < 0.05 vs. control (Cont) and vehicle (Veh)-treated animals, respectively; one-way ANOVA with post hoc Bonferroni’s multiple comparison).

PICK1 negatively regulates GRIA2 surface expression [21,22]. Src family and/or PKC-mediated GRIA2 phosphorylations facilitate GRIA2–PICK1 bindings, which lead to GRIA2 internalization and a relative increase of GRIA2-lacking, Ca^2+^-permeable AMPARs [21,23,24]. However, both perampanel and GYKI 52466 decrease GRIA1/GRIA2 ratio by reducing GRIA1, but not GRIA2, surface expression [19,20]. Considering GRIA2 phosphorylations, it is likely that altered PICK1 expression may be involved in unaffected GRIA2 surface expression by AMPAR antagonists. To confirm this, we evaluated the effects of perampanel and GYKI 52466 on PICK1 expression in the epileptic hippocampus. In the present study, PICK1 protein level was lower in chronic epilepsy rats than that in controls (*t*_(12)_ = 10.4, *p* < 0.001, Student *t*-test; Figure 6A,B and Figure S4), which was unaffected by AMPAR antagonists (*F*_(4,27)_ = 0.2, *p* = 0.93, one-way ANOVA; Figure 6A,B). These findings indicate that reduced PICK1 expression may decrease its binding to phosphorylated GRIA2, which would serve to maintain GRIA2 surface expression as an adaptive response. In addition, it is unlikely that PICK1-mediated GRIA2 internalization may be a contributor to generation of refractory seizures in response to AMPAR antagonists.

### 3.5. Effects of AMPAR Antagonists on GSK3β Phosphorylation

GSK3β is a serine/threonine kinase that phosphorylates various substrates and is involved in cellular and synaptic functions. Phosphoinositide 3-kinases (PI3K)/AKT-mediated S9 phosphorylation inhibits its activity, which is negatively regulated by PTEN [31,32,33]. GSK3β phosphorylates PICK1 and promotes the GRIA2–PICK1 interaction [34]. Furthermore, inactivated (phosphorylated) GSK3β promotes CREB S133 phosphorylation [32] that increases in human patients and animal models of epilepsy and participates in ictogenesis [35,36]. Furthermore, AMPAR antagonists activate PTEN-mediated CREB S133 dephosphorylation [19]. Since CREB is required for the maintenance of the GRIA1 surface expression under physiological conditions [37], it is likely that impaired AKT/GSK3β-mediated CREB S133 phosphorylation would result in refractory seizures in response to AMPAR antagonist.

In the present study, there was no difference in GSK3β protein level between control and the epileptic hippocampus (*t*_(12)_ = 1.1, *p* = 0.27, Student *t*-test; Figure 6C,D and Figure S5). Both perampanel and GYKI 52466 did not affect GSK3β protein level in responders and non-responders (*F*_(4,27)_ = 0.8, *p* = 0.53, one-way ANOVA; Figure 6C,D and Figure S5). However, GSK3β S9 phosphorylation level was 1.34-fold higher in the epileptic hippocampus than that in controls (*t*_(12)_ = 10.2, *p* < 0.001, Student *t*-test; Figure 6C,E and Figure S5) and its phosphorylation ratio was 1.39-fold of control level (*t*_(12)_ = 12.4, *p* < 0.001, Student *t*-test; Figure 6C,F). In responders, perampanel and GYKI 52466 restored GSK3β phosphorylation level to control level (*F*_(2,17)_ = 20.3, *p* < 0.001, one-way ANOVA; Figure 6C,E and Figure S5). Both AMPAR antagonists also recovered GSK3β phosphorylation ratio to control level (*F*_(2,17)_ = 27.5, *p* < 0.001, one-way ANOVA; Figure 6C,F). In non-responders, perampanel and GYKI 52466 did not affect GSK3β phosphorylation level (*F*_(2,16)_ = 0.5, *p* = 0.62, one-way ANOVA; Figure 6C,E) and its ratio (*F*_(2,16)_ = 0.7, *p* = 0.5, one-way ANOVA; Figure 6C,F). These findings indicate that AMPAR antagonists may liberate GSK3β from S9 phosphorylation-mediated inhibition.

**Figure 7:**
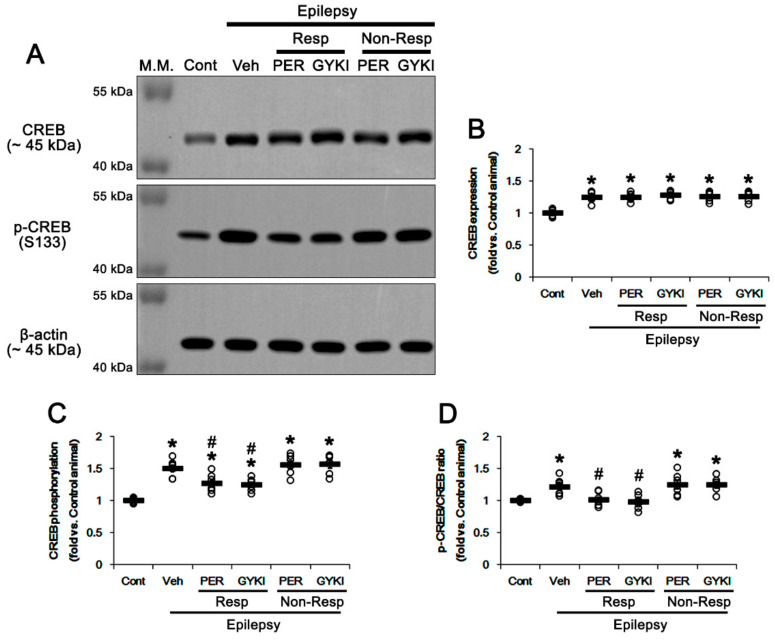
The effects of perampanel (PER) and GYKI 52466 (GYKI) on total Ca^2+^/cAMP response element-binding protein (CREB) expression and its S133 phosphorylation in chronic epilepsy rats. (**A**) Representative images for Western blot of CREB and its S133 phosphorylation level in the hippocampal tissues. (**B**–**D**) Quantifications of CREB (B), p-CREB S133 (**C**), and p-CREB/CREB ratio (**D**) in the hippocampal tissues. Open circles indicate each individual value. Horizontal bars indicate mean value. Error bars indicate SEM (*^,#^ *p* < 0.05 vs. control (Cont) and vehicle (Veh)-treated animals, respectively; one-way ANOVA with post hoc Bonferroni’s multiple comparison).

Consistent with our previous study [19], furthermore, CREB protein level in the epileptic hippocampus was 1.25-fold higher than that in controls (*t*_(12)_ = 7.1, *p* < 0.001, Student *t*-test; Figure 7A,B and Figure S6), which was unaffected by AMPAR antagonists (*F*_(4,27)_ = 0.2, *p* = 0.94, one-way ANOVA; Figure 7A,B). CREB S133 phosphorylation level was 1.51-fold higher in the epileptic hippocampus than that in controls (*t*_(12)_ = 9.8, *p* < 0.001, Student *t*-test; Figure 7A,C and Figure S6) and its phosphorylation ratio was 1.41-fold of control level (*t*_(12)_ = 6.9, *p* < 0.001, Student *t*-test; Figure 7A,D). In responders, perampanel and GYKI 52466 decreased CREB S133 phosphorylation level to 1.21- and 1.23-fold of control level, respectively (*F*_(2,17)_ = 9.1, *p* = 0.002, one-way ANOVA; Figure 7A,C). Both AMPAR antagonists reduced S133 phosphorylation ratios to control level (*F*_(2,17)_ = 7.7, *p* = 0.004, one-way ANOVA; Figure 7A,D). In non-responders, perampanel and GYKI 52466 did not affect CREB S133 phosphorylation level (*F*_(2,16)_ = 0.3, *p* = 0.75, one-way ANOVA; Figure 7A,C) and its ratio (*F*_(2,16)_ = 0.1, *p* = 0.89, one-way ANOVA; Figure 7A,D). These findings indicate that the GSK3β-CREB signaling pathway may be involved in anti-convulsive effects of AMPAR antagonists.

### 3.6. Effect of 3CAI Co-Treatment on Refractory Seizures in Non-Responders to AMPAR Antagonists

**Figure 8:**
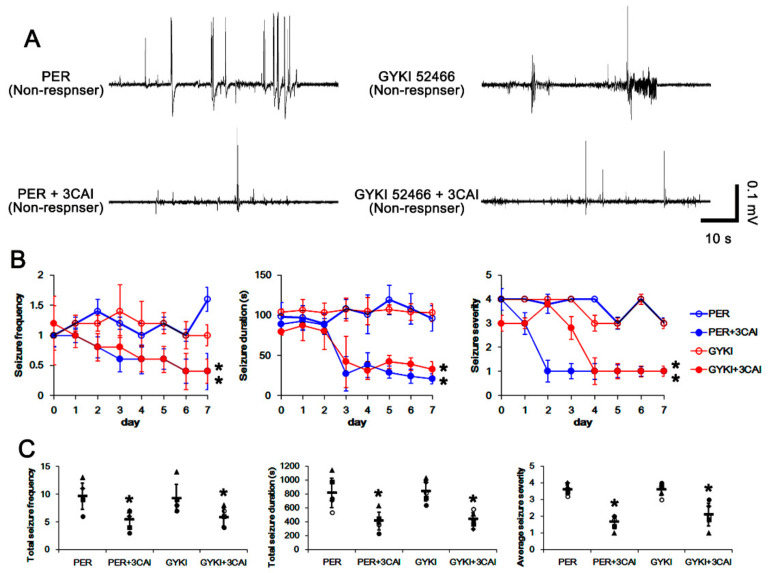
The effects of 3CAI co-treatment with perampanel (PER) and GYKI 52466 (GYKI) on spontaneous seizure activities in non-responders. 3CAI co-treatment effectively improves the anti-epileptic effects of both AMPAR antagonists in non-responders. (**A**) Representative electroencephalograms (EEG) in each group at 2 days after 3CAI co-treatment. (**B**) Quantitative analyses of the chronological effects of 3CAI co-treatment withAMPAR antagonists on seizure frequency, seizure duration, and seizure severity (seizure score) over 7-day period. Error bars indicate SD (* p < 0.05 vs. vehicle (Veh)-treated animals; Friedman test for seizure frequency and seizure severity; Repeated measures ANOVA for seizure duration). (**C**) Quantitative analyses of total seizure frequency, total seizure duration, and average behavioral seizure score (seizure severity) in 7-day period. Symbols indicate each individual value. Horizontal bars indicate mean value. Error bars indicate SD (* p < 0.05 vs. vehicle (Veh)-treated animals; Wilcoxon signed rank test for seizure frequency and seizure severity; paired Student *t*-test for seizure duration).

As mentioned earlier, GSK3β is inhibited by AKT-mediated S9 phosphorylation, which inhibits GRIA2–PICK1 interaction but promotes CREB S133 phosphorylation [31,32,33,34]. To confirm the roles of GSK3β signaling pathway in CREB activity and refractory seizures to AMPAR antagonists, 3CAI (an AKT inhibitor) was co-treated with perampanel or GYKI 52466 in non-responders (Figure 1). This is because the direct GSK3β activator is unavailable. In non-responders to perampanel, total seizure frequency was 9.6 ± 2.3, total seizure duration was 815.6 ± 213.7 s, and average seizure severity was 3.6 ± 0.3 over 1-week period (*n* = 5, Figure 8A–C). In non-responders to GYKI 52466, total seizure frequency was 9.2 ± 2.5, total seizure duration was 839 ± 144.1 s, and average seizure severity was 3.6 ± 0.4 over 1-week period (*n* = 5, Figure 8A–C). 3CAI co-treatment gradually decreased seizure frequency (*χ*^2^_(3)_ = 8.9, *p* = 0.031, Friedman test, *n* = 5), total seizure duration (*F*_(3,16)_ = 4.8, *p* = 0.014, repeated measures ANOVA, *n* = 5), and the seizure severity (*χ*^2^_(3)_ = 8.3, *p* = 0.041, Friedman test, *n* = 5) in both perampanel- and GYKI 52466-treated groups over 1-week period (Figure 8A,B). In non-responders to peramanel, 3CAI co-treatment reduced the total seizure frequency to 5.4 ± 1.6 (Z = 2.02, *p* = 0.04, Wilcoxon signed rank test, *n* = 5; Figure 8A,C), the total seizure duration to 409.2 ± 131.6 s (*t*_(4)_ = 3.12, *p* = 0.04, paired Student *t*-test, *n* = 5; Figure 8A,C), and average seizure severity to 1.6 ± 0.4 (Z = 2.04, *p* = 0.04, Wilcoxon signed rank test, *n* = 5; Figure 8A,C). In non-responders to GYKI 52466, 3CAI co-treatment also attenuated the seizure frequency to 5.8 ± 1.6 (Z = 2.03, *p* = 0.04, Wilcoxon signed rank test, *n* = 5; Figure 8A,C), the total seizure duration to 433.2 ± 100.1 s (*t*_(4)_ = 2.91, *p* = 0.04, paired Student *t*-test, *n* = 5; Figure 8A,C), and the seizure severity to 2.1 ± 0.7 (Z = 2.06, *p* = 0.04 vs. vehicle, Wilcoxon signed rank test, *n* = 5; Figure 8A,C). These findings indicate that PI3K/AKT-mediated GSK3β inhibition may be one of the important signaling pathways for generation of refractory seizures to AMPAR antagonists.

### 3.7. Effect of 3CAI Co-Treatment on PICK1 Expression and Phosphorylations of GSK3β and CREB in Non-Responders

**Figure 9:**
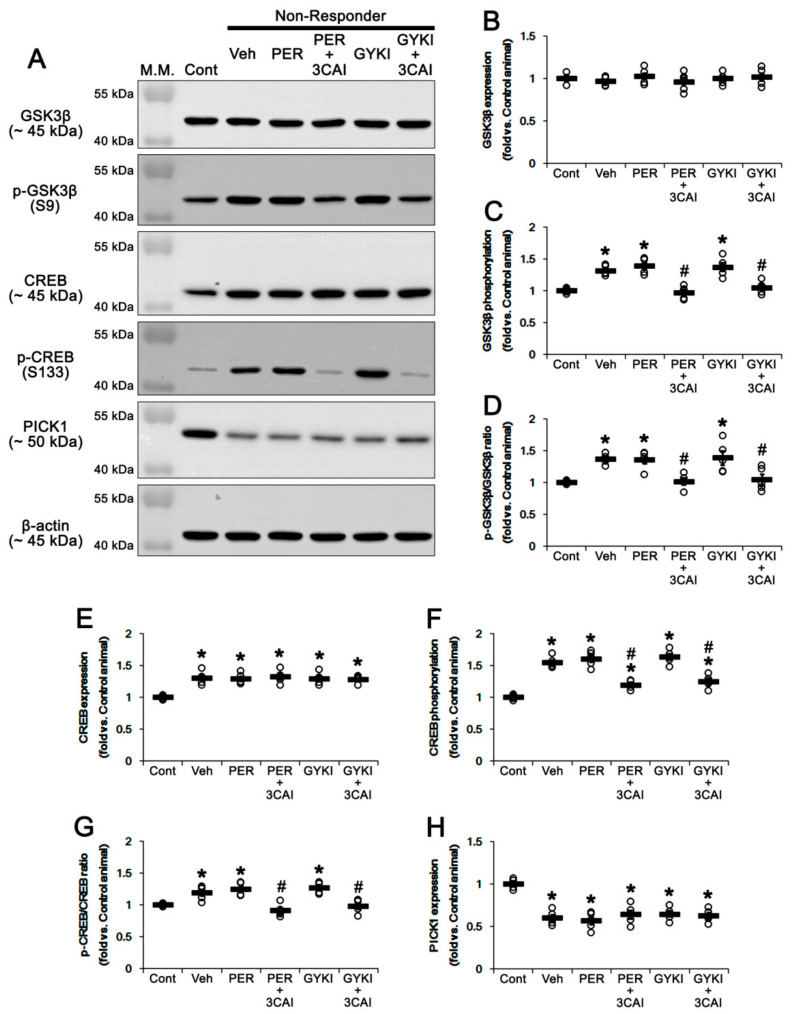
The effects of 3CAI co-treatment with perampanel (PER) and GYKI 52466 (GYKI) on expression levels of GSK3β, CREB, and PICK1 and the phosphorylation levels of GSK3β and CREB in non-responders. (**A**) Representative images for Western blot of total GSK3β, CREB, and PICK1 levels and the phosphorylation levels of GSK3β and CREB. (**B**–**H**) Quantifications of GSK3β (**B**), p-GSK3β S9 (**C**), p-GSK3β S9/GSK3β ratio (**D**), CREB (**E**), p-CREB S133 (**F**), p-CREB S133/CREB ratio (**G**), and PICK1 (**H**) in the hippocampal tissues. Open circles indicate each individual value. Horizontal bars indicate mean value. Error bars indicate SEM (*, ^#^ *p* < 0.05 vs. control (Cont) and vehicle (Veh)-treated animals, respectively; one-way ANOVA with post hoc Bonferroni’s multiple comparison).

Next, we investigated whether 3CAI co-treatment affects PICK1 expression and phosphorylations of GSK3β and CREB in non-responders to AMPAR antagonists. 3CAI co-treatment did not affect GSK3β protein level in non-responders (Figure 9A,B). However, 3CAI co-treatment reduced GSK3β S9 phosphorylation level to control level in non-responders to perampanel (*t*_(8)_ = 6.1, *p* < 0.001, Student *t*-test) and GYKI 52466 (*t*_(8)_ = 4.0, *p* = 0.004, Student *t*-test; Figure 9A,C and Figure S7). Thus, 3CAI co-treatment restored GSK3β S9 phosphorylation ratio to control level in non-responders to perampanel (*t*_(8)_ = 4.2, *p* = 0.003, Student *t*-test) and GYKI 52466 (*t*_(8)_ = 3.2, *p* = 0.01, Student *t*-test), respectively (Figure 9A,D). Similar to the case of GSK3β, 3CAI co-treatment did not alter CREB protein level in non-responders to perampanel and GYKI 52466 (Figure 9A,E and Figure S7). However, 3CAI co-treatment decreased CREB S133 phosphorylation level in non-responders to perampanel (*t*_(8)_ = 6.4, *p* < 0.001, Student *t*-test) and GYKI 52466 (*t*_(8)_ = 5.7, *p* < 0.001, Student *t*-test), respectively Figure 9A,F and Figure S7). 3CAI co-treatment restored CREB S133 phosphorylation ratio to control level in non-responders to perampanel (*t*_(8)_ = 5.4, *p* < 0.001, Student *t*-test) and GYKI 52466 (*t*_(8)_ = 4.9, *p* = 0.001, Student *t*-test), respectively (Figure 9A,G). 3CAI co-treatment did not alter PICK1 protein level in non-responders to perampanel and GYKI 52466 (Figure 9A,H). These findings indicate that the GSK3β activation may improve anti-convulsive effects of AMPAR antagonists in non-responders by regulating CREB activity rather than PICK1 expression.

### 3.8. Effect of 3CAI Co-Treatment on Surface GRIA Expressions in Non-Responders

**Figure 10:**
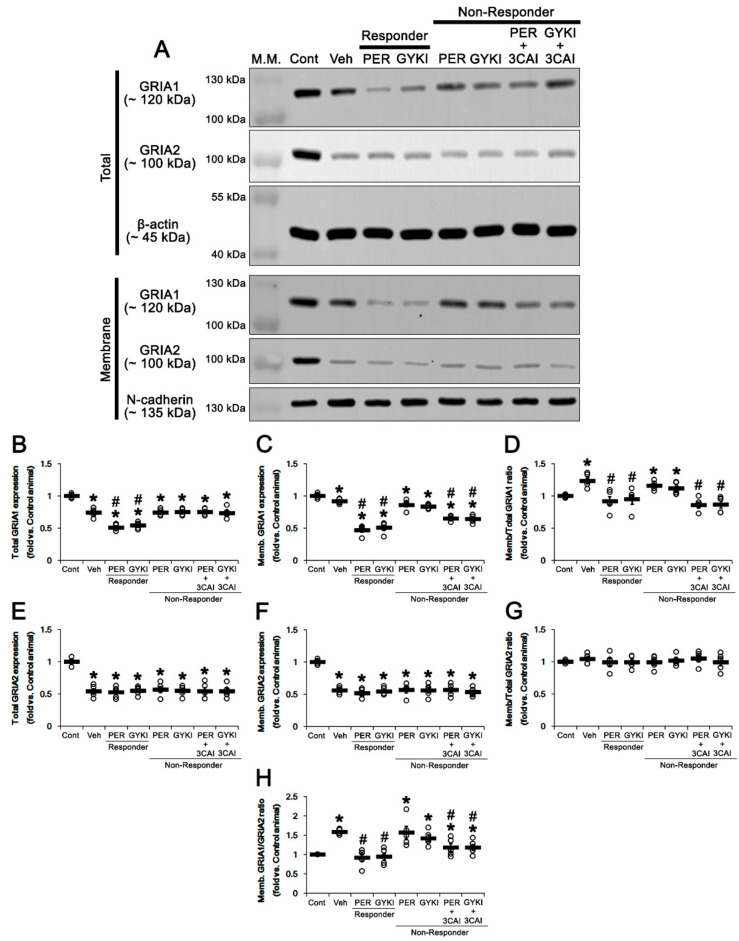
The effects of 3CAI co-treatment with perampanel (PER) and GYKI 52466 (GYKI) on surface expressions of GRIA1 and GRIA2 in chronic epilepsy rats. (**A**) Representative images for Western blot of total and membrane GRIA1 and GRIA2 expression levels. (**B**–**H**) Quantifications of total GRIA1 expression (**B**), membrane GRIA1 expression (**C**), membrane/total GRIA1 ratio (**D**), total GRIA2 expression (**E**), membrane GRIA2 expression (**F**), membrane/total GRIA2 ratio (**G**), and membrane GRIA1/GRIA2 ratio in the hippocampal tissues. Open circles indicate each individual value. Horizontal bars indicate mean value. Error bars indicate SEM (*, ^#^ *p* < 0.05 vs. control (Cont) and vehicle (Veh)-treated animals, respectively; one-way ANOVA with post hoc Bonferroni’s multiple comparison).

Finally, we investigated whether 3CAI co-treatment affects surface GRIA1 and GRIA2 expressions in the hippocampus of non-responders. The present study showed total GRIA1 protein level in the epileptic hippocampus was 0.74-fold of control level (*t*_(8)_ = 7.7, *p* < 0.001 vs. control animals, Student *t*-test; Figure 10A,B), which was further reduced to 0.51- and 0.54-fold of control level in responders by perampanel and GYKI 52466, respectively (*F*_(2,12)_ = 24.4, *p* < 0.001 vs. vehicle, one-way ANOVA; Figure 10A,B). However, both AMPAR antagonists did not influence total GRIA1 protein level in non-responders, which was unaffected by 3CAI co-treatment (Figure 10A,B and Figure S8).

Membrane GRIA1 expression in the epileptic hippocampus was 0.89-fold of control level (*t*_(8)_ = 4.1, *p* = 0.004 vs. control animals, Student *t*-test; Figure 10A,C). Thus, membrane/total GRIA1 ratio in the epileptic hippocampus was increased to 1.23-fold of control level (*t*_(8)_ = 3.4, *p* = 0.01 vs. control animals, Student *t*-test; Figure 10A,D and Figure S8). Perampanel and GYKI 52466 decreased membrane GRIA1 expression to 0.47- and 0.51-fold of control level in responders, respectively (*F*_(2,12)_ = 51.5, *p* < 0.001 vs. vehicle, one-way ANOVA; Figure 10A,C). Both AMPAR antagonists restored membrane/total GRIA1 ratio to control level in responders (*F*_(2,12)_ = 5.2, *p* = 0.02 vs. vehicle, one-way ANOVA; Figure 10A,D). Although both AMPAR antagonists did not affect membrane GRIA1 expression in non-responders, 3CAI co-treatment reduced it to 0.65- and 0.64-fold of control level in non-responders to perampanel (*t*_(8)_ = 5.7, *p* < 0.001, Student *t*-test) and GYKI 52466 (*t*_(8)_ = 6.4, *p* < 0.001, Student *t*-test), respectively (Figure 10A,C and Figure S8). 3CAI co-treatment also diminished membrane/total GRIA1 ratio to control level in non-responders to perampanel (*t*_(8)_ = 5.4, *p* < 0.001, Student *t*-test) and GYKI 52466 (*t*_(8)_ = 4.1, *p* = 0.003, Student *t*-test), respectively (Figure 10A,D). Both AMPAR antagonists and 3CAI co-treatment did not affect total GRIA2 protein level, membrane GRIA2 expression, and membrane/total GRIA2 ratio in both responders and non-responders (Figure 10A,E–G and Figure S8).

Membrane GRIA1/GRIA2 ratio in the epileptic hippocampus was 1.6-fold of control level (*t*_(8)_ = 18.8, *p* < 0.001 vs. control animals, Student *t*-test; Figure 10A,H and Figure S8). Both AMPAR antagonists restored membrane GRIA1/GRIA2 ratio to control level in responders (*F*_(2,12)_ = 23.1, *p* < 0.001 vs. vehicle, one-way ANOVA) but not non-responders (Figure 10A,H). 3CAI co-treatment decreased membrane GRIA1/GRIA2 ratio to 1.12- and 1.23-fold of control level in non-responders to perampanel (*t*_(8)_ = 2.9, *p* = 0.02, Student *t*-test) and GYKI 52466 (*t*_(8)_ = 3.2, *p* = 0.01, Student *t*-test), respectively (Figure 10A,H and Figure S8). These findings indicate that 3CAI may increase the responsiveness to AMPAR antagonists in non-responders by reducing surface GRIA1 expression.

## 4. Discussion

The principal findings of the present study were that AMPAR antagonists ameliorated spontaneous seizure activity by regulating surface expression of GRIA1 but not GRIA2. In addition, impaired AKT/GSK3β-mediated CREB S133 activation was involved in refractory seizures in response to AMPAR antagonists, which suggests that the regulation of these pathways may be a potential therapeutic strategy to improve the medication of intractable TLE.

Since the activation and desensitization kinetics of AMPAR are much faster than those of NMDAR [38], AMPAR antagonists have been focused to prevent ictogenesis and steadily progressive seizure-related brain pathologic plasticity in the epileptic brain [5]. Indeed, AMPAR antagonists terminate SE and seizure activity that are uncontrolled by NMDAR antagonists [6,7,8]. Among them, only perampanel is marketed for the treatment of focal epilepsy, which is a novel non-competitive AMPAR antagonist without affecting the NMDAR or kainate receptors [5], although its effects on signaling pathways remain to be elucidated. In the present study, both perampanel and GYKI 52466 ameliorated spontaneous seizure activity in 54% and 50% of animals (responders) in each group, concomitant with the reduced membrane expression of GRIA1 but not GRIA2. Furthermore, both AMPAR antagonists restored upregulated membrane/total GRIA1 ratio and membrane GRIA1/GRIA2 ratio to control levels. These effects of AMPAR antagonists were relevant to inhibition of AKT/GSK3β/CREB signaling pathway in responders. Because both AMPAR subunits are also located in endomembrane systems, such as the Golgi complex, endosomes, and ER [39,40], the GRIA1 and GRIA2 concentrations in membrane fraction may not directly represent the “pure surface expressions” in the present study. Considering that AMPAR trafficking is tightly regulated from the synthesis in ER to surface expression [41,42] and CREB regulates surface GRIA1 expression [37], however, the decreases in membrane/total GRIA1 ratio and membrane GRIA1/GRIA2 ratio indicate the diminished surface GRIA expression [19,20].

CREB activity (phosphorylation) is increased in human patients and animal models of epilepsy [35,36]. CREB phosphorylation is regulated by the activation of multiple signaling cascades, including GSK3β, PKC, protein kinase A (PKA), and Ca^2+^-calmodulin-dependent protein kinase II (CAMKII) [32,33,43,44]. In the present study, we found that GSK3β S9 (but not PKC) and CREB S133 phosphorylations were higher in the epileptic hippocampus than those in normal one, which were abolished by both AMPAR antagonists in responders, but not non-responders. Since the increased GSK3β-mediated CREB S133 phosphorylation participates in ictogenesis in human patients and animal models of epilepsy [32,35,36], these findings indicate that AMPAR antagonists may negatively regulate surface GRIA expression via GSK3β/CREB signaling pathway in responders.

PI3K/AKT-mediated GSK3β phosphorylation inhibits its activity, which is conversely regulated by PTEN [31,33]. Recently, we have reported that PTEN expression is decreased in the epileptic hippocampus, concomitant with the reduced Src/CK2-mediated phosphorylations [19]. Regarding phosphorylation-mediated inhibition of PTEN activity [45], we have postulated that the reduced PTEN phosphorylation (the increased activity) may be an adaptive response to the decreased expression in the epileptic hippocampus, while this alteration may be insufficient to suppress spontaneous seizure activities. This is because AMPAR antagonists further diminish PTEN phosphorylation only in responders, accompanied by increasing its expression, which is abrogated by dipotassium bisperoxovanadium(pic) dihydrate (BpV(pic), a PTEN inhibitor) [19,20]. In the present study, 3CAI-induced AKT inhibition improved the responsiveness to AMPAR antagonists in non-responders through GSK3β-mediated CREB regulation. Furthermore, 3CAI reduced surface GRIA1 expression in non-responders to AMPAR antagonists. Therefore, it is likely that the regulation of the PTEN/AKT/GSK3β/CREB pathway be essentially required for the anti-epileptic effects of AMPAR antagonists. Indeed, the hippocampi of animal models and the surgically resected hippocampal tissues from drug-resistant TLE patients show upregulated AKT activity (phosphorylation) [46,47,48]. Similar to the present data, furthermore, topiramate (TPM, an AED) modulates AMPAR-mediated AKT/GSK3β/CREB signaling pathway [49]. Therefore, our findings suggest that maladaptive regulation of AKT/GSK3β/CREB pathway may be one of the important causes in pharmacoresistant seizures in response to AMPAR antagonists.

PICK1 contributes to a shift in the AMPAR composition to GRIA2-lacking (Ca^2+^-permeable) by facilitating GRIA2 internalization [21,23,24]. In the present study, GRIA2 and PICK1 expression levels in the epileptic hippocampus were lower than those in the control (normal) hippocampus. These findings are consistent with a previous study demonstrating downregulation of GRIA2 and PICK1 expression in kainic acid-induced chronic epilepsy rats [17]. The present study also demonstrates that both AMPAR antagonists effectively reduced GRIA2 Y phosphorylations in responders, but not in non-responders, without affecting its S880 phosphorylation. Furthermore, GRIA2 Y phosphorylations showed direct proportional relationships with seizure parameters. Phosphorylations regulate surface GRIA2 expression through interactions with GRIP1 and PICK1: PKC-mediated S880 phosphorylation reinforces its internalization by inhibiting GRIP1–GRIA2 bindings [15,21]. In contrast, Src family-mediated Y phosphorylations facilitate PICK1–GRIA2 interaction to promote GRIA2 internalization [23,24]. Consistent with our previous studies [19,50], both AMPAR antagonists reduced Src family, but not PKC, activity. Considering the roles of GRIA2 phosphorylations in its interactions with GRIP1/PICK1 [15,21,23,24], it is plausible that both AMPAR antagonists would attenuate seizure activity by inhibiting PICK1-mediated GRIA2 internalization. However, AMPAR antagonists cannot influence surface GRIA2 expression [20]. In addition, the present data show that AMPAR antagonists did not influence the reduced PICK1 expression in the hippocampi of both responders and non-responders, which was unaffected by 3CAI co-treatment in non-responders, although PICK1 is a substrate of AKT/GSK3β pathway [34]. Therefore, it is likely that the reduced GRIA2 Y phosphorylations may reflect the decreased Src family activity induced by AMPAR antagonists rather than the inhibition of GRIA2-lacking AMPAR assembly. Furthermore, it is hypothesized that reduced PICK1 expression in the hippocampus would be involved in ictogenesis in chronic epilepsy rats via other pathways rather than the regulation of surface GRIA2 expression since PICK1 interacts with type III metabotropic glutamate receptor (mGlu7R) in the presynaptic active zone and regulates glutamate release [50]. Indeed, mGlu7R deletion and a polypeptide interfering PICK1 and mGlu7R show typical epileptic seizures [51,52]. Therefore, our findings indicate that PICK1-mediated regulation of surface GRIA2 expression may not be involved in the anti-epileptic effects and responsiveness of AMPAR antagonists, although both perampanel and GYKI 52466 inhibited Src-mediated GRIA2 Y phosphorylations.

On the other hand, SE and spontaneous seizure activity lead to vasogenic edema formation (serum extravasation) induced by brain–blood barrier (BBB) disruption, which deteriorates seizure activity [53,54,55]. During recovery of vasogenic edema, multidrug efflux transporter expressions, such as p-glycoprotein (p-GP), breast cancer resistance protein (BCRP), and multidrug resistance protein-4 (MRP4), are increased in the hippocampus, which reduce AED concentrations in the brain and cause pharmacoresistant epilepsy [53]. Since we did not compare the pharmacokinetics of AMPAR antagonists between responders and non-responders in the present study, the possibility could not be excluded that the decreased responsiveness of AMPAR antagonists in non-responders would be a consequence from the lower concentration of these compounds induced by over-expression or hyper-activation of drug efflux transporters. In addition, AKT is one of the common down-stream molecules during seizure-induced BBB leakage. Indeed, 3CAI effectively attenuates SE-induced vasogenic edema formation, although it does not show anti-epileptic effects [28]. Thus, it is likely that 3CAI would also increase the efficacies of AMPAR antagonist by inhibiting AKT-mediated serum leakage or upregulation of multidrug efflux transporter. On the other hand, CREB activation plays a protective role against BBB disruption [56,57]. Considering the inhibitory effect of 3CAI on AKT/GSK3β/CREB signaling pathway in the present study, however, it is unlikely that 3CAI may be involved in the CREB-mediated regulation of vascular permeability. Further studies are needed to elucidate whether 3CAI enhances the responsiveness to AMPAR antagonists by inhibiting serum extravasation and/or multidrug efflux systems.

## 5. Conclusions

The present study revealed that AMPAR antagonists ameliorated spontaneous seizure activity by affecting the Src-mediated AKT/GSK3β/CREB signaling pathway, which was relevant to the regulation of surface expression of GRIA1 rather than GRIA2. In addition, the dysregulation of this pathway was one of the causes of refractory seizures to AMPAR antagonists. Therefore, our findings suggest that the Src/AKT/GSK3β/CREB pathway may be one of the potential therapeutic targets for the treatment of intractable TLE.

## Figures and Tables

**Table 1:** Primary antibodies used in the present study.

Antigen	Host	Manufacturer (Catalog Number)	Dilution Used
CREB	Rabbit	Novus biologicals (NBP1-90364)	1:500 (WB)
GRIA1	Mouse	Synaptic systems (#182011)	1:1000 (WB)
GRIA2	Rabbit	Sigma (AB1768-I)	1:1000 (WB)
GSK3β	Rabbit	Elapscience (ENT2082)	1:1000 (WB)
N-cadherin	Rabbit	Abcam (ab182030)	1:4000 (WB)
p-CREB S133	Rabbit	Novus biologicals (NB110-55727)	1:5000 (WB)
p-GRIA2 Y869/Y873/Y876	Rabbit	Cell signaling (#3921)	1:1000 (WB)
p-GRIA2 S880	Rabbit	Invitrogen (#PA5-38134)	1:1000 (WB)
p-GSK3β S9	Rabbit	Biorbyt (orb14745)	1:1000 (WB)
p-PKC T497	Rabbit	Abcam (ab76016)	1:1000 (WB)
p-Src family Y416	Rabbit	Cell signaling (#6943)	1:1000 (WB)
p-Src family Y527	Rabbit	Cell signaling (#2105)	1:1000 (WB)
PICK1	Rabbit	Abcam (ab3420)	1:1000 (WB)
PKC	Rabbit	Abcam (ab23511)	1:1000 (WB)
Src family	Rabbit	Cell signaling (#2108)	1:1000 (WB)
β-actin	Mouse	Sigma (#A5316)	1:5000 (WB)

## Supplementary Materials

The following are available online at https://www.mdpi.com/2227-9059/9/4/425/s1, Figure S1: Representative full-gel images of Western blots in Figure 3A, Figure S2: Representative full-gel images of Western blots in Figure 4A, Figure S3: Representative full-gel images of Western blots in Figure 5A, Figure S4: Representative full-gel images of Western blots in Figure 6A, Figure S5: Representative full-gel images of Western blots in Figure 6B, Figure S6: Representative full-gel images of Western blots in Figure 7A, Figure S7: Representative full-gel images of Western blots in Figure 9A, Figure S8: Representative full-gel images of Western blots in Figure 10A.

## References

[1] Aylward R.L. (2008). Epilepsy: A review of reports, guidelines, recommendations and models for the provision of care for patients with epilepsy. Clin. Med..

[2] Mohanraj R., Norrie J., Stephen L.J., Kelly K., Hitiris N., Brodie M.J. (2006). Mortality in adults with newly diagnosed and chronic epilepsy: A retrospective comparative study. Lancet Neurol..

[3] Blair R.E., Deshpande L.S., Sombati S., Elphick M.R., Martin B.R., DeLorenzo R.J. (2009). Prolonged exposure to WIN55,212-2 causes downregulation of the CB1 receptor and the development of tolerance to its anticonvulsant effects in the hippocampal neuronal culture model of acquired epilepsy. Neuropharmacology.

[4] Blair R.E., Sombati S., Churn S.B., Delorenzo R.J. (2008). Epileptogenesis causes an N-methyl-d-aspartate receptor/Ca2+-dependent decrease in Ca2+/calmodulin-dependent protein kinase II activity in a hippocampal neuronal culture model of spontaneous recurrent epileptiform discharges. Eur. J. Pharmacol..

[5] Roberta C., Francesco F. (2020). Targeting ionotropic glutamate receptors in the treatment of epilepsy. Curr. Neuropharmacol..

[6] Fritsch B., Stott J.J., Joelle Donofrio J., Rogawski M.A. (2010). Treatment of early and late kainic acid-induced status epilepticus with the noncompetitive AMPA receptor antagonist GYKI 52466. Epilepsia.

[7] Niquet J., Baldwin R., Norman K., Suchomelova L., Lumley L., Wasterlain C.G. (2017). Simultaneous triple therapy for the treatment of status epilepticus. Neurobiol. Dis..

[8] Mohammad H., Sekar S., Wei Z., Moien-Afshari F., Taghibiglou C. (2019). Perampanel but not amantadine prevents behavioral alterations and epileptogenesis in pilocarpine rat model of status epilepticus. Mol. Neurobiol..

[9] Essin K., Nistri A., Magazanik L. (2002). Evaluation of GluR2 subunit involvement in AMPA receptor function of neonatal rat hypoglossal motoneurons. Eur. J. Neurosci..

[10] Greger I.H., Khatri L., Ziff E.B. (2002). RNA editing at arg607 controls AMPA receptor exit from the endoplasmic reticulum. Neuron.

[11] Barria A., Derkach V., Soderling T. (1997). Identification of the Ca2+/calmodulin-dependent protein kinase II regulatory phosphorylation site in the alpha-amino-3-hydroxyl-5-methyl-4-isoxazole-propionate-type glutamate receptor. J. Biol. Chem..

[12] Seidenman K.J., Steinberg J.P., Huganir R., Malinow R. (2003). Glutamate receptor subunit 2 Serine 880 phosphorylation modulates synaptic transmission and mediates plasticity in CA1 pyramidal cells. J. Neurosci..

[13] Lee H.K., Barbarosie M., Kameyama K., Bear M.F., Huganir R.L. (2000). Regulation of distinct AMPA receptor phosphorylation sites during bidirectional synaptic plasticity. Nature.

[14] Malinow R., Malenka R.C. (2002). AMPA receptor trafficking and synaptic plasticity. Annu. Rev. Neurosci..

[15] Wyszynski M., Valtschanoff J.G., Naisbitt S., Dunah A.W., Kim E., Standaert D.G., Weinberg R., Sheng M. (1999). Association of AMPA receptors with a subset of glutamate receptor-interacting protein in vivo. J. Neurosci..

[16] Ma Y., Sun X., Li J., Jia R., Yuan F., Wei D., Jiang W. (2017). Melatonin alleviates the epilepsy-associated impairments in hippocampal LTP and spatial learning through rescue of surface GluR2 expression at hippocampal CA1 synapses. Neurochem. Res..

[17] Lorgen J.Ø., Egbenya D.L., Hammer J., Davanger S. (2017). PICK1 facilitates lasting reduction in GluA2 concentration in the hippocampus during chronic epilepsy. Epilepsy Res..

[18] Pellegrini-Giampietro D.E., Gorter J.A., Bennett M.V., Zukin R.S. (1997). The GluR2 (GluR-B) hypothesis: Ca(2+)-permeable AMPA receptors in neurological disorders. Trends Neurosci..

[19] Kim J.E., Lee D.S., Park H., Kang T.C. (2020). Src/CK2/PTEN-mediated GluN2B and CREB dephosphorylations regulate the responsiveness to AMPA receptor antagonists in chronic epilepsy Rats. Int. J. Mol. Sci..

[20] Kim J.E., Park H., Lee J.E., Kim T.H., Kang T.C. (2020). PTEN is required for the anti-epileptic effects of AMPA receptor antagonists in chronic epileptic rats. Int. J. Mol. Sci..

[21] Chung H.J., Xia J., Scannevin R.H., Zhang X., Huganir R.L. (2000). Phosphorylation of the AMPA receptor subunit GluR2 differentially regulates its interaction with PDZ domain-containing proteins. J. Neurosci..

[22] Kim C.H., Chung H.J., Lee H.K., Huganir R.L. (2001). Interaction of the AMPA receptor subunit GluR2/3 with PDZ domains regulates hippocampal long-term depression. Proc. Natl. Acad. Sci. USA.

[23] Ahmadian G., Ju W., Liu L., Wyszynski M., Lee S.H., Dunah A.W., Taghibiglou C., Wang Y., Lu J., Wong T.P. (2004). Tyrosine phosphorylation of GluR2 is required for insulin-stimulated AMPA receptor endocytosis and LTD. EMBO J..

[24] Hayashi T., Huganir R.L. (2004). Tyrosine phosphorylation and regulation of the AMPA receptor by SRC family tyrosine kinases. J. Neurosci..

[25] Ko A.R., Kang T.C. (2015). Blockade of endothelin B receptor improves the efficacy of levetiracetam in chronic epileptic rats. Seizure.

[26] Racine R.J. (1972). Modification of seizure activity by electrical stimulation. II. Motor seizure. Electroencephalogr. Clin. Neurophysiol..

[27] Kim J.E., Choi H.C., Song H.K., Kang T.C. (2019). Perampanel affects up-stream regulatory signaling pathways of GluA1 phosphorylation in normal and epileptic rats. Front. Cell. Neurosci..

[28] Kim J.E., Park H., Lee J.E., Kang T.C. (2020). Blockade of 67-kDa laminin receptor facilitates AQP4 down-regulation and BBB disruption via ERK1/2-and p38 MAPK-mediated PI3K/AKT activations. Cells.

[29] Roskoski R. (2005). Src kinase regulation by phosphorylation and dephosphorylation. Biochem. Biophys. Res. Commun..

[30] Roskoski R. (2004). Src protein-tyrosine kinase structure and regulation. Biochem. Biophys. Res. Commun..

[31] Takashima A. (2009). Drug development targeting the glycogen synthase kinase-3beta (GSK-3beta)-mediated signal transduction pathway: Role of GSK-3beta in adult brain. J. Pharmacol. Sci..

[32] Grimes C.A., Jope R.S. (2001). CREB DNA binding activity is inhibited by glycogen synthase kinase-3 beta and facilitated by lithium. J. Neurochem..

[33] Grimes C.A., Jope R.S. (2001). The multifaceted roles of glycogen synthase kinase 3beta in cellular signaling. Prog. Neurobiol..

[34] Yagishita S., Murayama M., Ebihara T., Maruyama K., Takashima A. (2015). Glycogen synthase kinase 3β-mediated phosphorylation in the most C-terminal region of protein interacting with C kinase 1 (PICK1) regulates the binding of PICK1 to glutamate receptor subunit GluA2. J. Biol. Chem..

[35] Becker A.J., Chen J., Zien A., Sochivko D., Normann S., Schramm J., Elger C.E., Wiestler O.D., Blümcke I. (2003). Correlated stage- and subfield-associated hippocampal gene expression patterns in experimental and human temporal lobe epilepsy. Eur. J. Neurosci..

[36] Zhu X., Dubey D., Bermudez C., Porter B.E. (2015). Suppressing cAMP response element-binding protein transcription shortens the duration of status epilepticus and decreases the number of spontaneous seizures in the pilocarpine model of epilepsy. Epilepsia.

[37] Middei S., Houeland G., Cavallucci V., Ammassari-Teule M., D’Amelio M., Marie H. (2013). CREB is necessary for synaptic maintenance and learning-induced changes of the AMPA receptor GluA1 subunit. Hippocampus.

[38] Paoletti P. (2011). Molecular basis of NMDA receptor functional diversity. Eur. J. Neurosci..

[39] Parkinson G.T., Hanley J.G. (2018). Mechanisms of AMPA Receptor Endosomal Sorting. Front. Mol. Neurosci..

[40] Moretto E., Passafaro M. (2018). Recent Findings on AMPA Receptor Recycling. Front. Cell. Neurosci..

[41] Greger I.H., Esteban J.A. (2007). AMPA receptor biogenesis and trafficking. Curr. Opin. Neurobiol..

[42] Zhu J.J. (2003). Mechanisms of synaptic plasticity: From membrane to intracellular AMPAR trafficking. Mol. Interv..

[43] Brindle P.K., Montminy M.R. (1992). The CREB family of transcription activators. Curr. Opin. Genet. Dev..

[44] Sassone-Corsi P. (1995). Transcription factors responsive to cAMP. Annu. Rev. Cell Dev. Biol..

[45] Moult P.R., Cross A., Santos S.D., Carvalho A.L., Lindsay Y., Connolly C.N., Irving A.J., Leslie N.R., Harvey J. (2010). Leptin regulates AMPA receptor trafficking via PTEN inhibition. J. Neurosci..

[46] Shacka J.J., Lu J., Xie Z.L., Uchiyama Y., Roth K.A., Zhang J. (2007). Kainic acid induces early and transient autophagic stress in mouse hippocampus. Neurosci. Lett..

[47] Zhu F., Kai J., Chen L., Wu M., Dong J., Wang Q., Zeng L.H. (2018). Akt Inhibitor perifosine prevents epileptogenesis in a rat model of temporal lobe epilepsy. Neurosci. Bull..

[48] Talos D.M., Jacobs L.M., Gourmaud S., Coto C.A., Sun H., Lim K.C., Lucas T.H., Davis K.A., Martinez-Lage M., Jensen F.E. (2018). Mechanistic target of rapamycin complex 1 and 2 in human temporal lobe epilepsy. Ann. Neurol..

[49] Shalaby H.N., El-Tanbouly D.M., Zaki H.F. (2018). Topiramate mitigates 3-nitropropionic acid-induced striatal neurotoxicity via modulation of AMPA receptors. Food Chem. Toxicol..

[50] Rothstein J.D., Martin L., Levey A.I., Dykes-Hoberg M., Jin L., Wu D., Nash N., Kuncl R.W. (1994). Localization of neuronal and glial glutamate transporters. Neuron.

[51] Arstikaitis P., Gauthier-Campbell C. (2006). BARS at the synapse: PICK-1 lipid binding domain regulates targeting, trafficking, and synaptic plasticity. J. Neurosci..

[52] Bertaso F., Zhang C., Scheschonka A., de Bock F., Fontanaud P., Marin P., Huganir R.L., Betz H., Bockaert J., Fagni L. (2008). PICK1 uncoupling from mGluR7a causes absence-like seizures. Nat. Neurosci..

[53] Kim Y.J., Kim J.E., Choi H.C., Song H.K., Kang T.C. (2015). Cellular and regional specific changes in multidrug efflux transporter expression during recovery of vasogenic edema in the rat hippocampus and piriform cortex. BMB Rep..

[54] Kim J.E., Kang T.C. (2017). TRPC3- and ET_B_ receptor-mediated PI3K/AKT activation induces vasogenic edema formation following status epilepticus. Brain Res..

[55] Broekaart D.W.M., Anink J.J., Baayen J.C., Idema S., de Vries H.E., Aronica E., Gorter J.A., van Vliet E.A. (2018). Activation of the innate immune system is evident throughout epileptogenesis and is associated with blood-brain barrier dysfunction and seizure progression. Epilepsia.

[56] Ruan W., Li J., Xu Y., Wang Y., Zhao F., Yang X., Jiang H., Zhang L., Saavedra J.M., Shi L. (2019). MALAT1 up-regulator polydatin protects brain microvascular integrity and ameliorates stroke through C/EBPβ/MALAT1/CREB/PGC-1α/PPARγ pathway. Cell. Mol. Neurobiol..

[57] Wu X., Fu S., Liu Y., Luo H., Li F., Wang Y., Gao M., Cheng Y., Xie Z. (2019). NDP-MSH binding melanocortin-1 receptor ameliorates neuroinflammation and BBB disruption through CREB/Nr4a1/NF-κB pathway after intracerebral hemorrhage in mice. J. Neuroinflamm..

