# Supplementary material for: Inhibition of AKT/GSK3β/CREB Pathway Improves the Responsiveness to AMPA Receptor Antagonists by Regulating GRIA1 Surface Expression in Chronic Epilepsy Rats
Source: Biomedicines. 2021 Apr 14;9(4):425. doi: 10.3390/biomedicines9040425 (PMC8103519; doi:10.3390/biomedicines9040425)
Supplement: Supplementary file 1 [file biomedicines-09-00425-s001.pdf]

Supplementary information

Inhibition of AKT/GSK3β/CREB pathway improves the responsiveness to AMPA receptor antagonists by regulating GRIA1 surface expression in chronic epilepsy rats

Ji-Eun Kim, Duk-Shin Lee, Hana Park, Tae-Hyun Kim, Tae-Cheon Kang[*]

Department of Anatomy and Neurobiology and Institute of Epilepsy Research, College of Medicine, Hallym University, Chuncheon 24252, Korea

***** Correspondence: Department of Anatomy and Neurobiology and Institute of Epilepsy Research, College of Medicine, Hallym University, Chuncheon 24252, Korea. E-mail: tckang@hallym.ac.kr; Tel.:+82-33-248-2524; Fax: +82-33-248-2525.

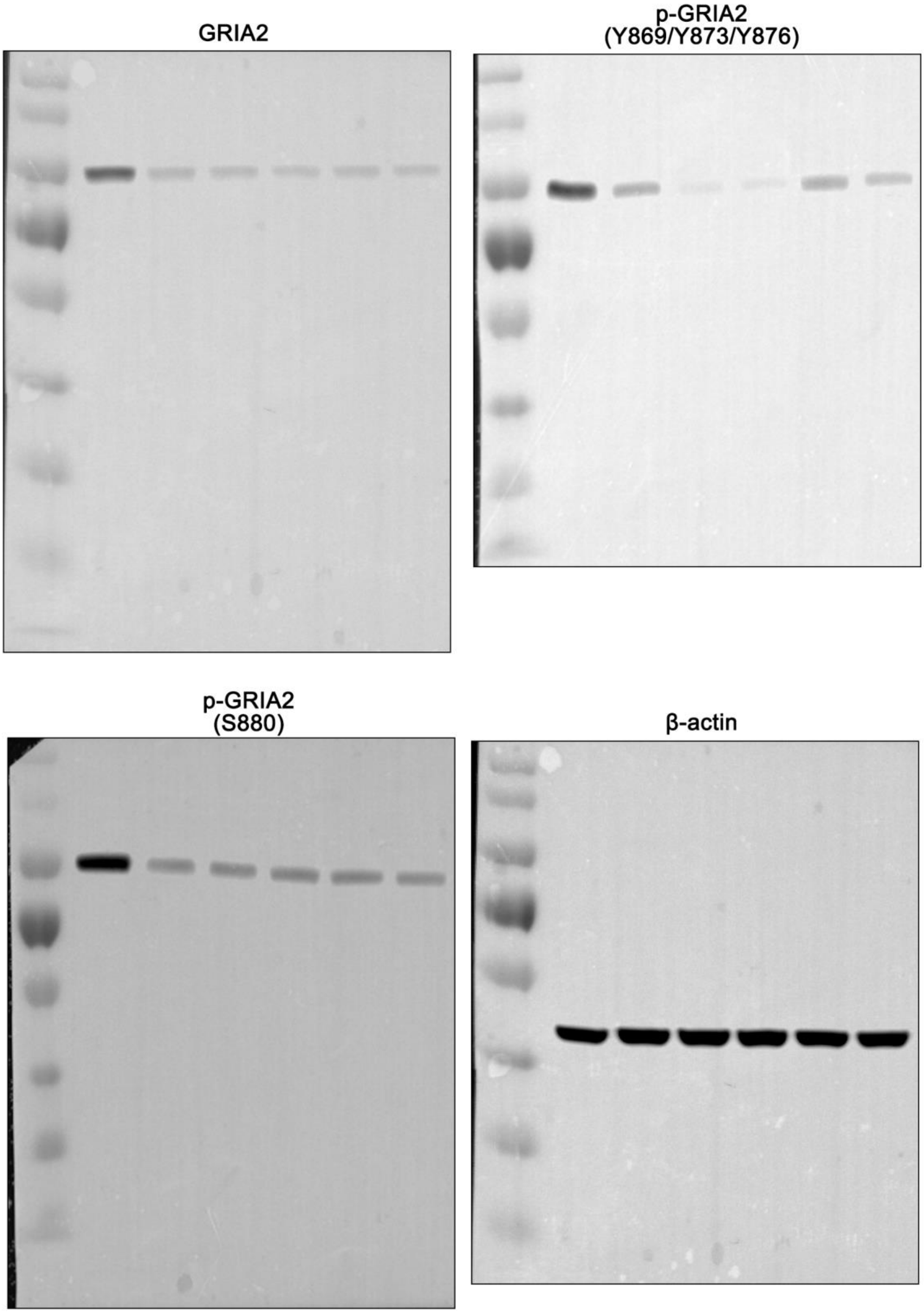

Supplementary Figure 1. Representative full-gel images of Western blots in Figure 3A.

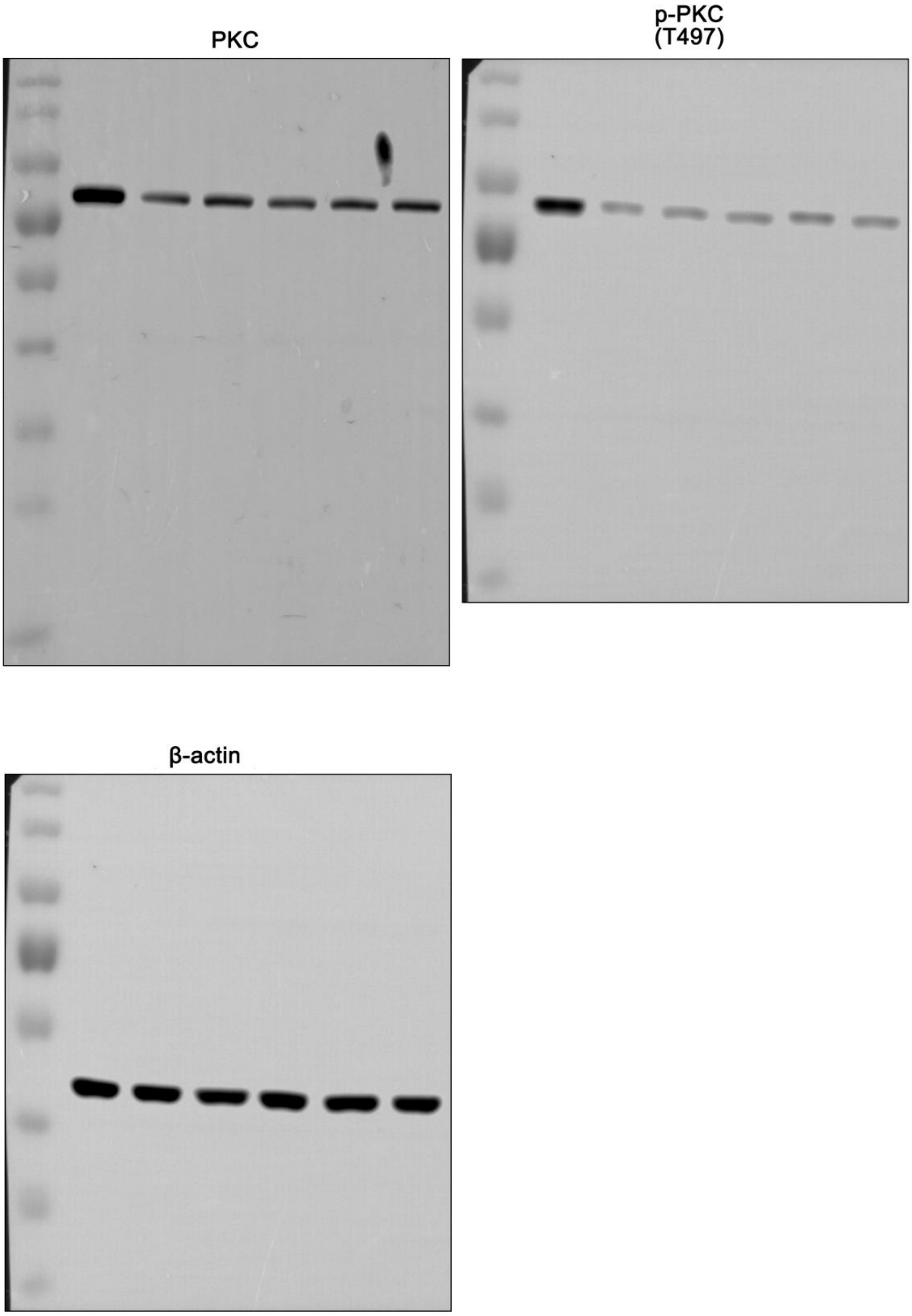

Supplementary Figure 2. Representative full-gel images of Western blots in Figure 4A.

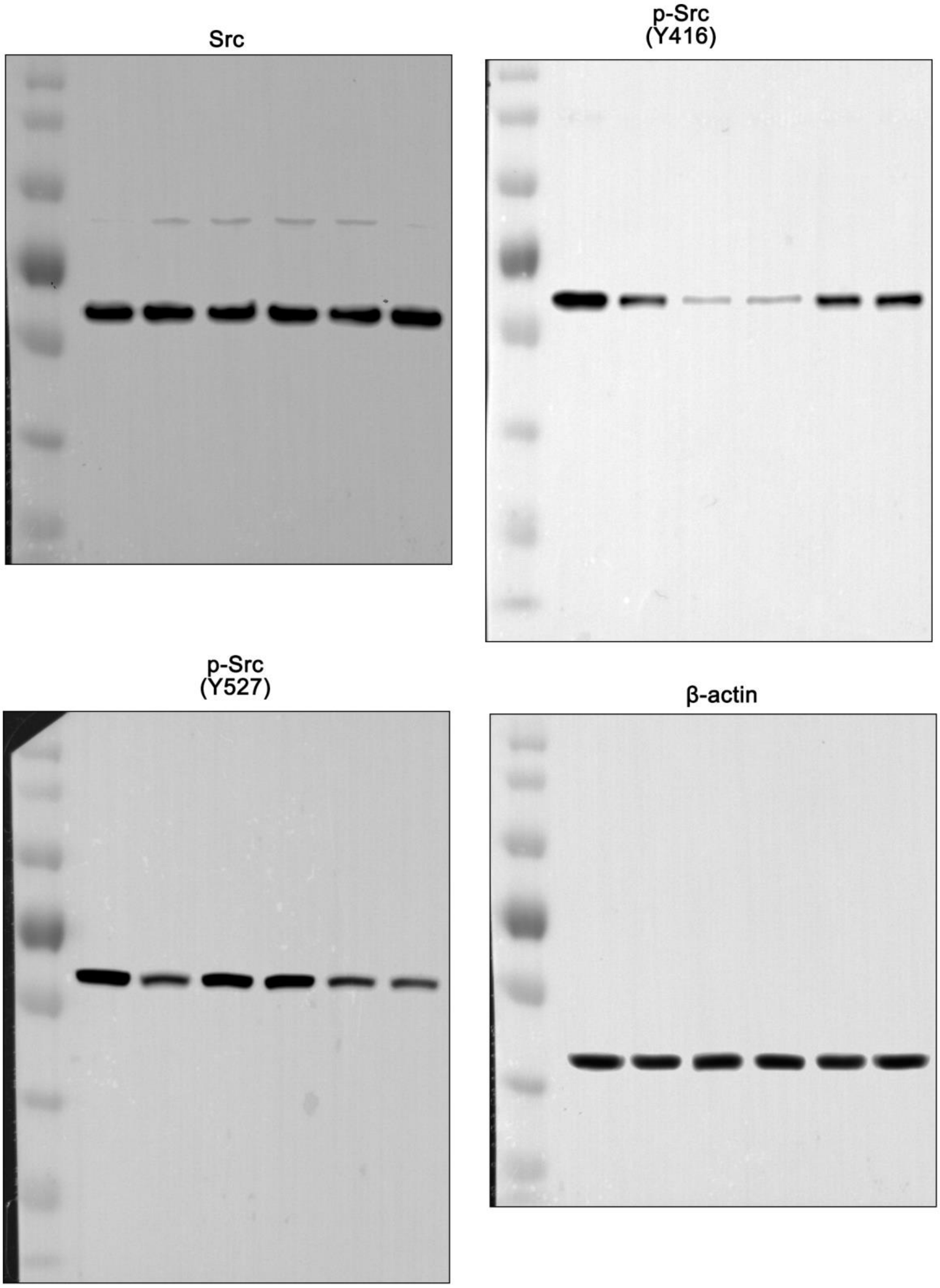

Supplementary Figure 3. Representative full-gel images of Western blots in Figure 5A.

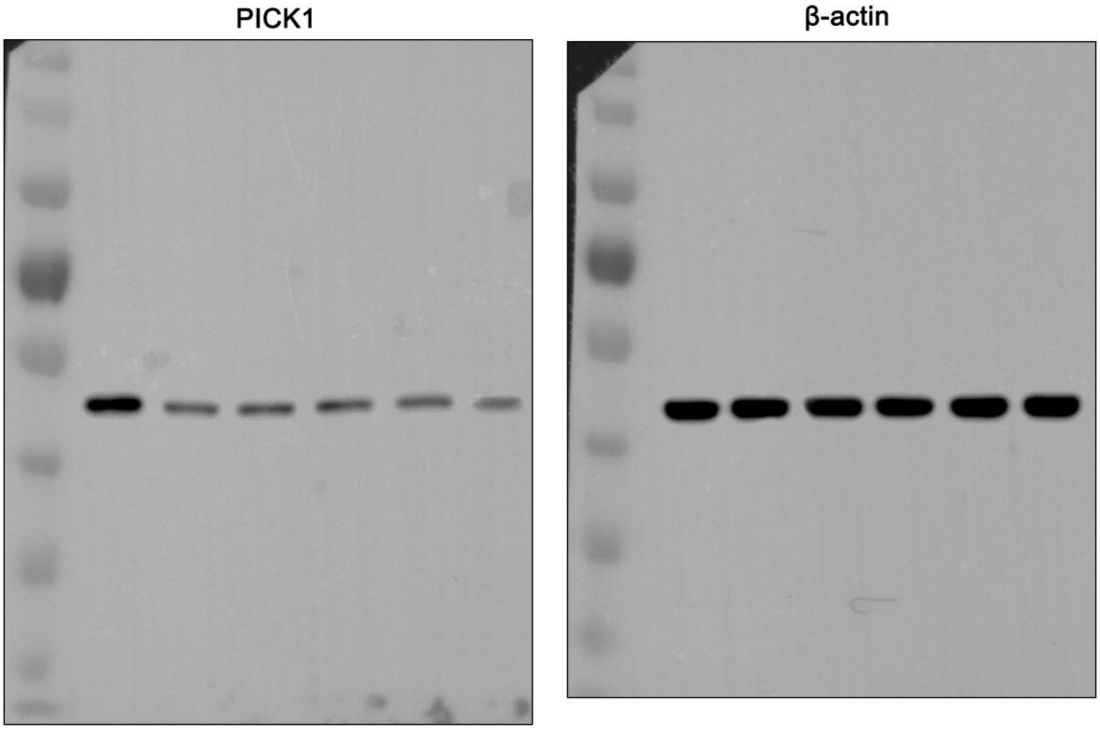

Supplementary Figure 4. Representative full-gel images of Western blots in Figure 6A.

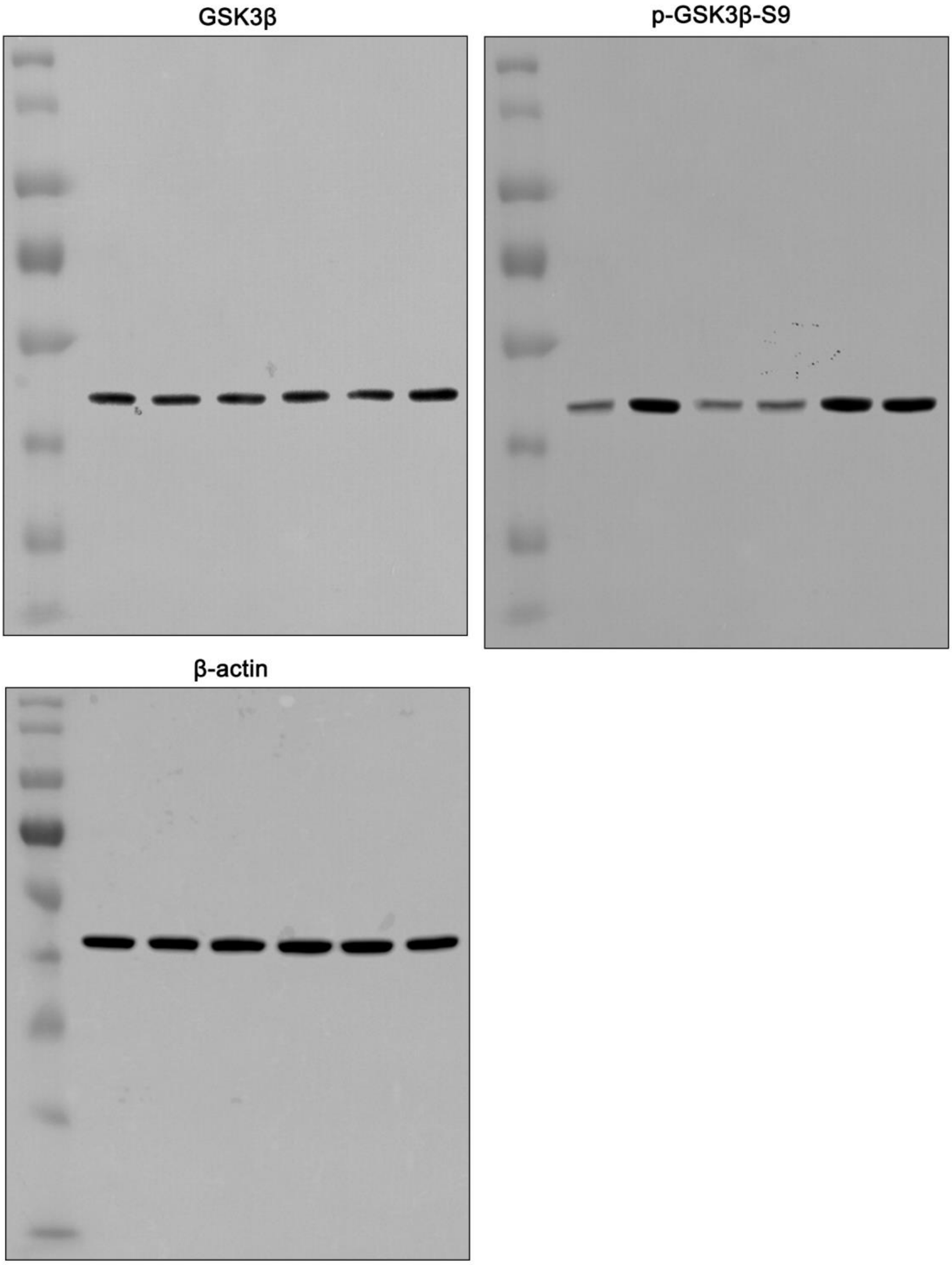

Supplementary Figure 5. Representative full-gel images of Western blots in Figure 6B.

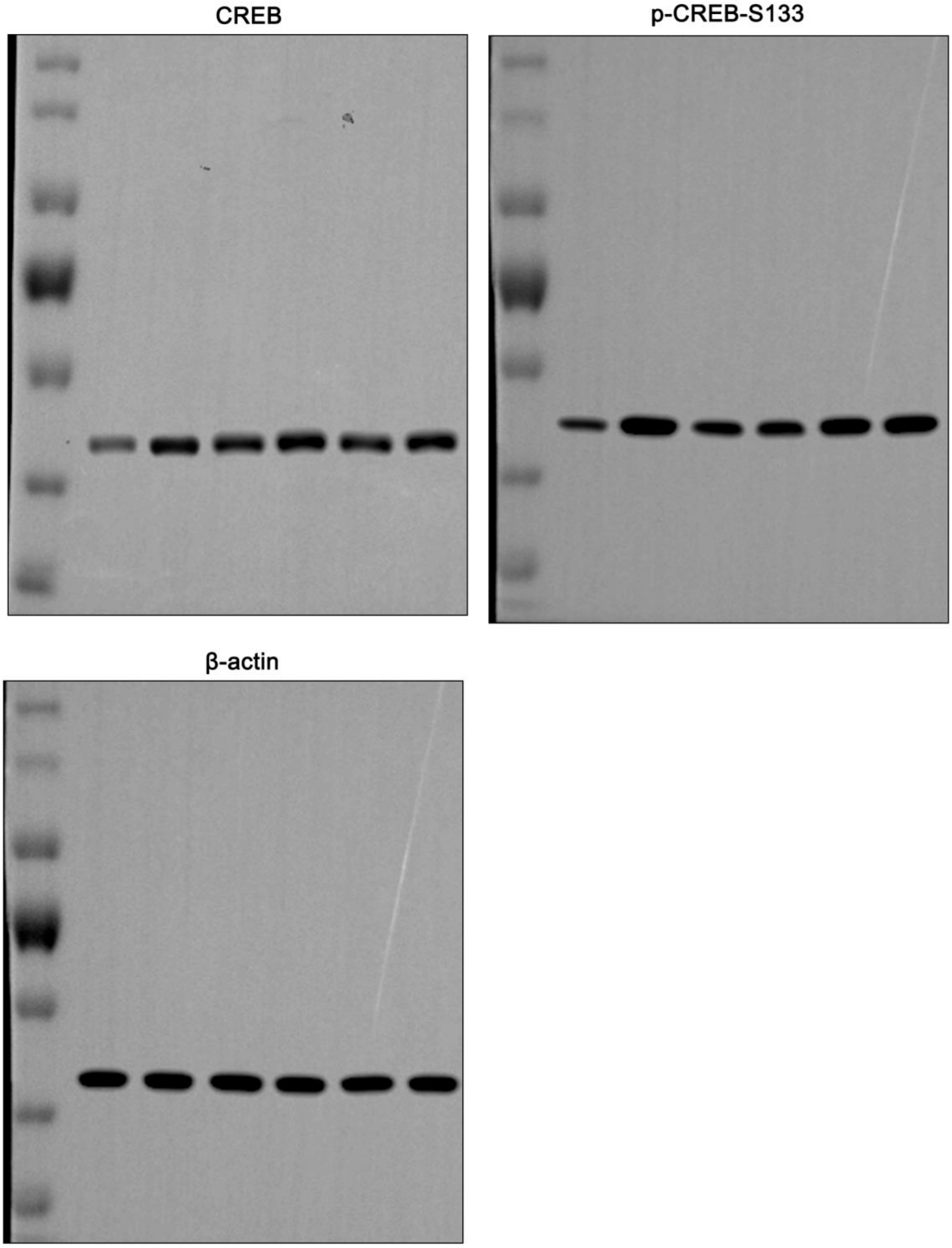

Supplementary Figure 6. Representative full-gel images of Western blots in Figure 7A.

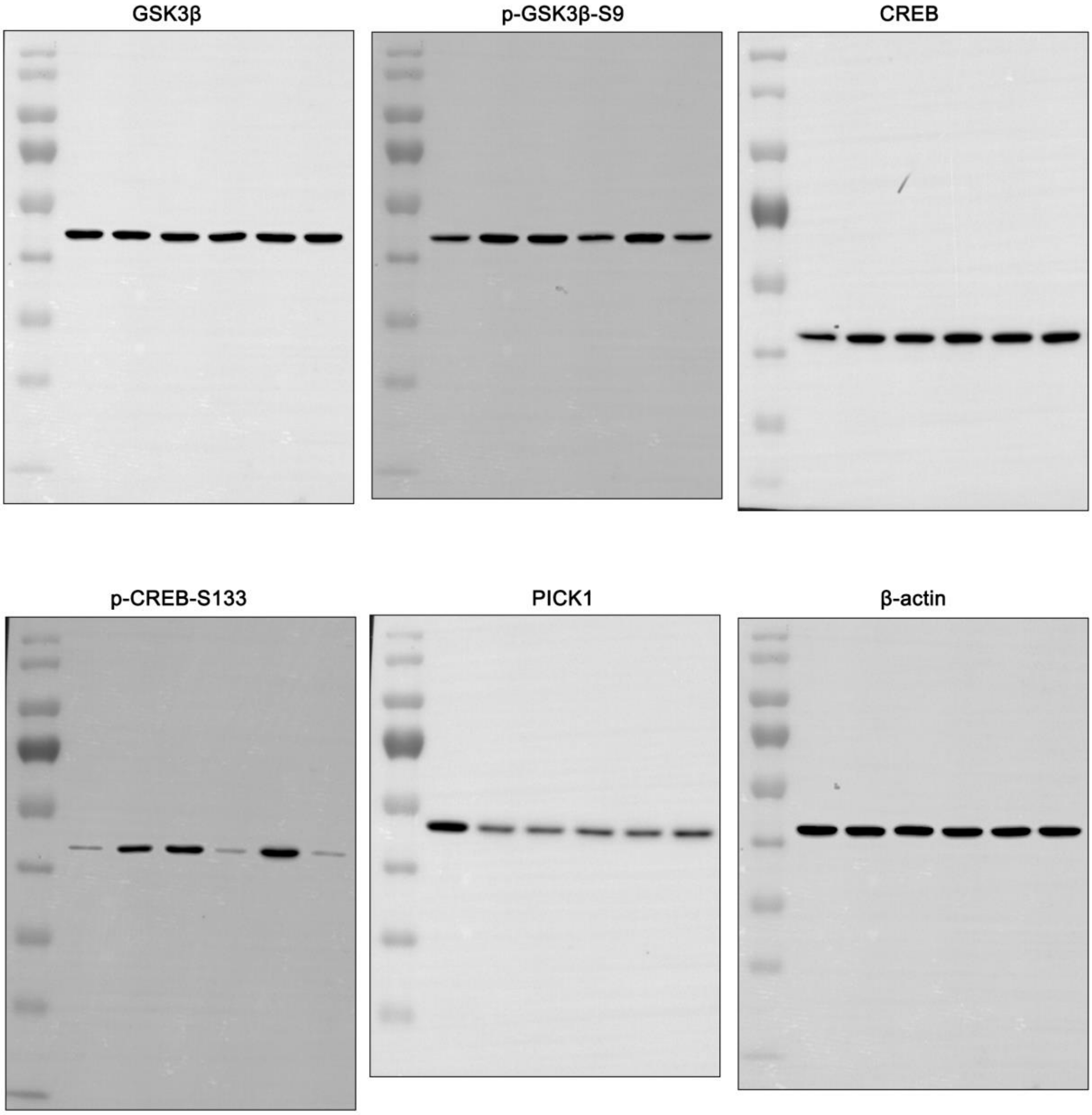

Supplementary Figure 7. Representative full-gel images of Western blots in Figure 9A.

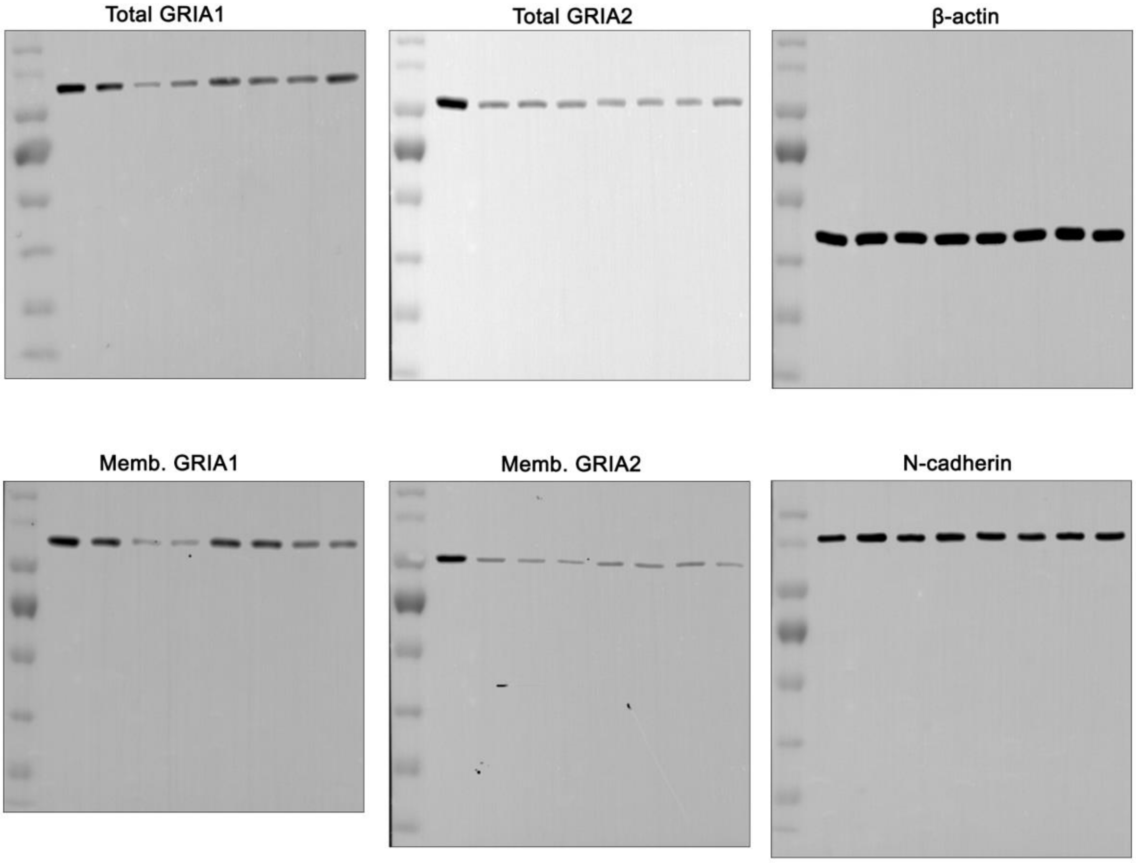

Supplementary Figure 8. Representative full-gel images of Western blots in Figure 10A.
